# Myosin XVI Regulates Actin Cytoskeleton Dynamics in Dendritic Spines of Purkinje Cells and Affects Presynaptic Organization

**DOI:** 10.3389/fncel.2019.00330

**Published:** 2019-08-13

**Authors:** Mona Katrin Roesler, Franco Luis Lombino, Sandra Freitag, Michaela Schweizer, Irm Hermans-Borgmeyer, Jürgen R. Schwarz, Matthias Kneussel, Wolfgang Wagner

**Affiliations:** ^1^Department of Molecular Neurogenetics, Center for Molecular Neurobiology Hamburg, University Medical Center Hamburg-Eppendorf, Hamburg, Germany; ^2^Electron Microscopy Unit, Center for Molecular Neurobiology Hamburg, University Medical Center Hamburg-Eppendorf, Hamburg, Germany; ^3^Transgenic Animal Unit, Center for Molecular Neurobiology Hamburg, University Medical Center Hamburg-Eppendorf, Hamburg, Germany

**Keywords:** Purkinje cell, dendritic spine, actin cytoskeleton, autism spectrum disorder, *Myo16*, WAVE, Arp2/3, synaptic vesicles

## Abstract

The actin cytoskeleton is crucial for function and morphology of neuronal synapses. Moreover, altered regulation of the neuronal actin cytoskeleton has been implicated in neuropsychiatric diseases such as autism spectrum disorder (ASD). Myosin XVI is a neuronally expressed unconventional myosin known to bind the WAVE regulatory complex (WRC), a regulator of filamentous actin (F-actin) polymerization. Notably, the gene encoding the myosin’s heavy chain (*MYO16*) shows genetic association with neuropsychiatric disorders including ASD. Here, we investigated whether myosin XVI plays a role for actin cytoskeleton regulation in the dendritic spines of cerebellar Purkinje cells (PCs), a neuronal cell type crucial for motor learning, social cognition and vocalization. We provide evidence that both myosin XVI and the WRC component WAVE1 localize to PC spines. Fluorescence recovery after photobleaching (FRAP) analysis of GFP-actin in cultured PCs shows that *Myo16* knockout as well as PC-specific *Myo16* knockdown, lead to faster F-actin turnover in the dendritic spines of PCs. We also detect accelerated F-actin turnover upon interference with the WRC, and upon inhibition of Arp2/3 that drives formation of branched F-actin downstream of the WRC. In contrast, inhibition of formins that are responsible for polymerization of linear actin filaments does not cause faster F-actin turnover. Together, our data establish myosin XVI as a regulator of the postsynaptic actin cytoskeleton and suggest that it is an upstream activator of the WRC-Arp2/3 pathway in PC spines. Furthermore, ultra-structural and electrophysiological analyses of *Myo16* knockout cerebellum reveals the presence of reduced numbers of synaptic vesicles at presynaptic terminals in the absence of the myosin. Therefore, we here define myosin XVI as an F-actin regulator important for presynaptic organization in the cerebellum.

## Introduction

Synaptic development, function and plasticity depend on a functional neuronal actin cytoskeleton (Cingolani and Goda, 2008; Hotulainen and Hoogenraad, 2010; Konietzny et al., 2017). F-actin is enriched at presynaptic terminals and within postsynaptic dendritic spines, small cellular protrusions on which excitatory synapses are found (Matus et al., 1982; Cingolani and Goda, 2008; Honkura et al., 2008; Korobova and Svitkina, 2010; Rust and Maritzen, 2015). F-actin comprises a meshwork of branched and linear filaments and provides structural support to dendritic spines. Moreover, it serves as track for actin-based myosin motors, some of which deliver cargos needed for postsynaptic plasticity (Wagner et al., 2011a; Kneussel and Wagner, 2013). A large fraction of the actin cytoskeleton in dendritic spines is highly dynamic and undergoes continuous turnover (“treadmilling”), i.e., cycles of nucleation of new filaments, their elongation by polymerization, filament severing and depolymerization (Star et al., 2002; Pollard and Borisy, 2003; Chazeau and Giannone, 2016; Konietzny et al., 2017). Importantly, the regulation of actin dynamics appears to be fundamental for processes associated with learning and memory such as structural and functional synaptic plasticity (Matus, 2000; Honkura et al., 2008; Kasai et al., 2010; MacGillavry et al., 2013; Rust and Maritzen, 2015; Hlushchenko et al., 2016; Basu and Lamprecht, 2018; Borovac et al., 2018).

Myosin XVI is a neuronally expressed, vertebrate-specific unconventional myosin (Patel et al., 2001; Yokoyama et al., 2011; Cameron et al., 2013). There are indications that the myosin might be involved in actin cytoskeleton regulation. The myosin XVI heavy chain (MYO16, also known as NYAP3) is an F-actin-binding protein with an N-terminal ankyrin repeat domain that binds protein phosphatase 1 catalytic subunits, followed by a myosin motor domain that, in mammals, is likely impaired in its ability to hydrolyze ATP (Patel et al., 2001; Cameron et al., 2007; Kengyel et al., 2015). Via its tail domain, MYO16 binds phosphoinositide 3-kinase (PI3K) and the WRC, an upstream regulator of Arp2/3-dependent actin filament nucleation (Yokoyama et al., 2011). MYO16/NYAP3 and two proteins that resemble the myosin’s tail domain (NYAP1, NYAP2) are thought to function redundantly via bridging WRC-PI3K association in order to regulate neurite outgrowth (Yokoyama et al., 2011). Notably, both WRC and Arp2/3 are key factors that drive actin dynamics in hippocampal dendritic spines (Spence and Soderling, 2015; Chazeau and Giannone, 2016).

The WRC constitutes a heteropentameric complex consisting of WAVE1 (or its orthologs WAVE2, WAVE3), CYFIP1 (also known as SRA1; or its ortholog CYFIP2/PIR121), NCKAP1 (also known as NAP1, HEM2; or its ortholog HEM1), ABI1 (or its orthologs ABI2, ABI3) and HSPC300 (also known as BRICK1) (Takenawa and Suetsugu, 2007; Bisi et al., 2013). Activation of the WRC is a multistep process that involves binding to the small GTPase Rac1, and results in exposure of the VCA domain of WAVE (Lebensohn and Kirschner, 2009; Chen et al., 2010). Once exposed, the VCA domain binds and activates Arp2/3. The seven subunit Arp2/3 complex catalyzes the nucleation of new filaments from the side of pre-existing ones, thereby promoting formation of a branched F-actin meshwork (Rotty et al., 2013).

Genetic approaches demonstrated the importance of WRC- and Arp2/3-mediated actin dynamics for synaptic structure and function. Ablation of WRC components leads to abnormal F-actin turnover in hippocampal spines and to changes in spine density and morphology (Grove et al., 2004; Kim Y. et al., 2006; Hazai et al., 2013; Pathania et al., 2014; Njoo et al., 2015). Loss of WAVE1 furthermore causes deficits in synaptic plasticity, learning, and memory (Soderling et al., 2003, 2007). Moreover, genetic disruption of Arp2/3 alters F-actin dynamics in hippocampal spines, structural spine plasticity, and AMPA receptor recruitment into synapses (Hotulainen et al., 2009; Kim et al., 2013; Spence et al., 2016). Many more factors are known that control actin dynamics in hippocampal spines and impact synaptic plasticity, including further Arp2/3 regulators (Mikhaylova et al., 2018; Parkinson et al., 2018), non-muscle myosin IIb (Rex et al., 2010; Koskinen et al., 2014), and post-translational modification of actin subunits (Bertling et al., 2016). Finally, formins have been detected at the tip of finger-like protrusions growing out from hippocampal spine heads (Hotulainen et al., 2009; Chazeau et al., 2014). Similar to Arp2/3, formins drive *de novo* formation of actin filaments that are, however, linear and lead to the formation of elongated protrusions such as filopodia.

Interestingly, several genes linked to an increased risk of developing ASD encode actin regulators (Joensuu et al., 2018). This includes the genes encoding WRC components CYFIP1 and NCKAP1, two established myosin XVI protein interaction partners (Wang et al., 2009; Yokoyama et al., 2011; Chang et al., 2013; Joensuu et al., 2018). ASD is a complex neuropsychiatric disease characterized by deficits in social interaction and communication, with motor coordination problems as a frequent comorbidity (Wang et al., 2014; de la Torre-Ubieta et al., 2016). Strikingly, ASD-like phenotypes in mouse models can be reverted by manipulating actin regulators (Dolan et al., 2013; Duffney et al., 2015). Therefore, it has been suggested that alterations in F-actin dynamics are one of the important pathological features in ASD (Spence and Soderling, 2015; Lin et al., 2016; Yan et al., 2016; Borovac et al., 2018; Hlushchenko et al., 2018; Joensuu et al., 2018). Notably, genetic evidence links also *MYO16* to an increased risk of developing ASD (Wang et al., 2009; Chang et al., 2013; Liu et al., 2015) and other neuropsychiatric disorders (Rodriguez-Murillo et al., 2014; Kao et al., 2016).

MYO16 occurs in cerebellar PCs (Patel et al., 2001; Cameron et al., 2013), central signal integrators that provide the only output from the cerebellar cortex. Their dendrites project into the cerebellar molecular layer and receive excitatory synaptic input via dendritic spines from axons termed PFs (granule cell axons) and climbing fibers (CFs). PCs are crucial for motor coordination and motor learning (Ito, 2001; Schonewille et al., 2010). However, recent research demonstrates that PCs are also important for social cognition, language and vocalization (Tsai et al., 2012; Fujita-Jimbo and Momoi, 2014; Peter et al., 2016; Sokolov et al., 2017). Interestingly, malfunction of the cerebellum and PCs has been linked to ASD and ASD-like phenotypes in mice (Wang et al., 2014; de la Torre-Ubieta et al., 2016). For example, PC-specific knockout of ASD genes *Shank2* or *Tsc1* causes social interaction deficits in mice (Tsai et al., 2012; Peter et al., 2016). Thus, cerebellar PCs appear to be a highly relevant cell type for studying the role of ASD-related genes such as *Myo16*.

Notably, little is known about F-actin regulation in the dendritic spines of PCs, compared to hippocampal neurons. PCs express WRC components, the formin Daam1, and the Arp2/3-formin coordinator MTSS1 (Soderling et al., 2003; Saarikangas et al., 2015; Kawabata Galbraith et al., 2018). However, whether and how the WRC, Arp2/3, and formins affect F-actin turnover in PC spines has not been examined directly. Remarkably, PCs also harbor specific F-actin regulators such as espin and delphilin that are not found in other neurons (Miyagi et al., 2002; Sekerkova et al., 2003). Thus, actin dynamics might be regulated in a unique manner in PC dendritic spines.

The aim of the present study was to obtain insight into the role of myosin XVI in neuronal cells. Given the known interaction of MYO16 with the actin regulator WRC, and considering that several genes associated with ASD encode actin regulators, we hypothesized that myosin XVI is involved in regulating dendritic spine F-actin and, possibly, synaptic structure and function.

## Results

### Generation and Initial Characterization of *Myo16* Knockout Mice

**FIGURE 1:**
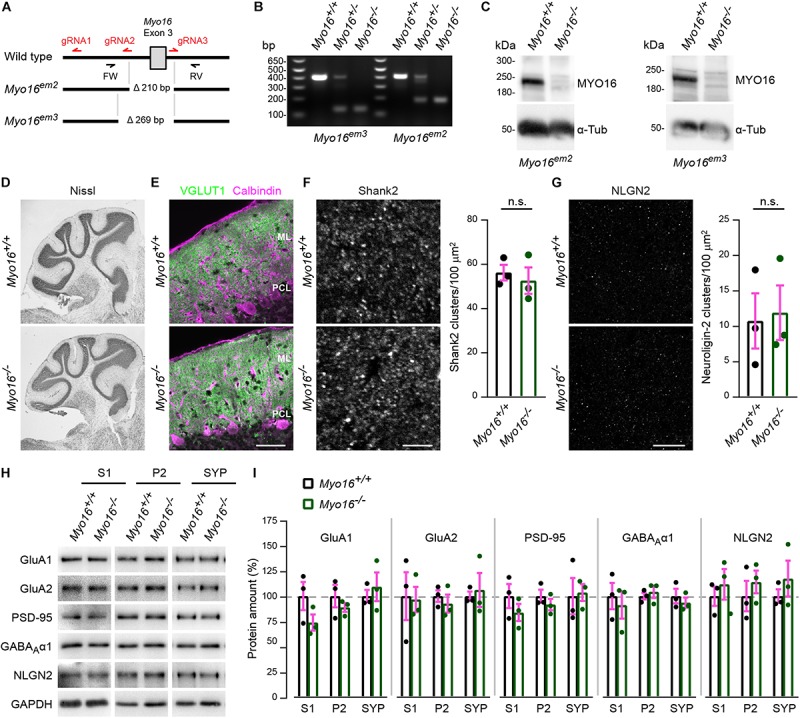
Generation and initial characterization of *Myo16* knockout mice. **(A)** Overview of CRISPR/Cas9-mediated creation of *Myo16* knockout mice (see section “Materials and Methods” for details). Guide RNA sequences are indicated in red (gRNA1-3). Mouse lines carrying deletions of 210 bp (*Myo16^*em*2^*) and 269 bp (*Myo16^*em*3^*) were obtained, with both deletions comprising exon 3 of *Myo16*. Genotyping primers indicated (FW, RV). **(B)** Identification of mice that are hetero- or homozygous carriers of the *Myo16^*em*3^* and *Myo16^*em*2^* alleles via PCR genotyping using FW and RV primers. Shown is an agarose gel on which the obtained DNA fragments were separated. **(C)** Absence of MYO16 protein in cerebellum extracts of homozygous *Myo16^*em*2^* and *Myo16^*em*3^* mice. Western blot analysis was performed using cerebellar extracts from 11 day old wild-type (*Myo16^+/+^*) and homozygous knockout (*Myo16^–/–^*) mice. Antibodies against MYO16 (pre-adsorbed as described in section “Materials and Methods”) and α-Tubulin (α-Tub; loading control) were used. **(D)** Nissl staining of sagittal cerebellar sections from adult wild-type (*Myo16^+/+^*) and *Myo16^*em*3^* knockout (*Myo16^–/–^*) mice. **(E)** Sagittal cerebellar sections from adult wild-type (*Myo16^+/+^*) and *Myo16^*em*3^* knockout (*Myo16^–/–^*) mice immuno-fluorescently labeled with antibodies against VGLUT1 (PF terminal marker) and Calbindin-D-28K (Purkinje cell marker). ML, molecular layer; PCL, Purkinje cell layer. Scale bar, 50 μm. **(F)** Sagittal sections of the cerebellar molecular layer from adult *Myo16^+/+^* and *Myo16^*em*3^* knockout mice (*Myo16^–/–^*) were immuno-fluorescently labeled with antibodies against Shank2. Graph depicts number of Shank2 clusters per 100 μm^2^ as mean ± SEM (magenta), *n* = 3 (single data points represent mice analyzed), no significant differences detected (n.s.; Student’s *t*-test; see section “Materials and Methods”). Scale bar, 5 μm. **(G)** Sagittal sections of the cerebellar molecular layer from adult *Myo16^+/+^* and *Myo16^*em*3^* knockout mice (*Myo16^–/–^*) were immuno-fluorescently labeled with antibodies against neuroligin-2 (NLGN2). Graph depicts number of NLGN2 clusters per 100 μm^2^ as mean ± SEM (magenta), *n* = 3 (single data points represent mice analyzed), no significant differences detected (n.s.; Student’s *t*-test). Scale bar, 20 μm. **(H,I)** Cerebellar extracts of 25 week old *Myo16^+/+^* and *Myo16^*em*3^* knockout mice (*Myo16^–/–^*) were subjected to subcellular fractionation. S1, P2, and SYP fractions were analyzed by Western blot using the indicated antibodies. In **(I)**, quantification of the indicated proteins from Western blots of S1, P2, and SYP fractions of *Myo16^+/+^* and *Myo16^–/–^* mice is shown. Graphs depict protein amount after normalization to GAPDH loading control, as percentage of the mean protein amount in the respective *Myo16^+/+^* fraction. Bars represent mean values (*n* = 3; data points represent mice analyzed) ± SEM (magenta), no significant differences were detected when comparing protein amounts between the same fraction (S1, P2, or SYP) of *Myo16^+/+^* and *Myo16^–/–^* mice (n.s.; Student’s *t*-test).

To investigate the role of MYO16 in the brain, we generated two mouse lines that carry constitutive *Myo16* knockout alleles (*Myo16^*em*2^*, *Myo16^*em*3^*; Figures 1A,B). Western blot analyses confirmed the absence of myosin XVI heavy chain in homozygous *Myo16^–/–^* mice of both lines (Figure 1C). Since *Myo16* is expressed in cerebellum (Patel et al., 2001; Cameron et al., 2007; see also Figure 1C), we examined the anatomical organization of this structure in *Myo16^–/–^* knockout mice. Nissl staining of cerebellar sections did not reveal gross abnormalities regarding foliation and the organization of layers (Figure 1D). Moreover, immuno-fluorescence labeling for the presynaptic marker VGLUT1 did not expose gross deficits in terms of presence of PF terminals in the molecular layer (Figure 1E). Given the genetic association of *MYO16* with ASD, we also examined the *in situ* localization of Shank2 and neuroligin-2, two proteins that are strongly linked to ASD and that form postsynaptic clusters at excitatory and inhibitory synapses of PCs, respectively (Zhang et al., 2015; Ha et al., 2016; Peter et al., 2016). Quantification of Shank2 clusters (Figure 1F) and neuroligin-2 clusters (Figure 1G) within the cerebellar molecular layer showed that cluster density is unaltered in the absence of myosin XVI. Finally, we characterized protein levels and subcellular distribution of postsynaptic molecules in *Myo16^–/–^* knockout cerebellum (Figures 1H,I). We focused on excitatory synapse proteins (AMPA receptor subunits GluA1 and GluA2, scaffolding molecule PSD-95) and on inhibitory synapse proteins (GABA_A_ receptor subunit α1, neuroligin-2). All of these proteins are present in cerebellum, including postsynaptically in PCs (Briatore et al., 2010; Yamasaki et al., 2011; Zhang et al., 2015). Using differential fractionation of cerebellar extracts, we generated a crude extract (S1), a membrane-enriched fraction (P2) and a fraction enriched for synaptosomal proteins (SYP) (Figure 1H). Quantification of GluA1, GluA2, PSD-95, GABA_A_ α1, and neuroligin-2 amounts showed that the levels of these synaptic proteins in S1, P2, and SYP fractions are not significantly changed in the absence of myosin XVI, compared to littermate control (Figure 1I). Together, this indicates that gross anatomical organization, as well as expression and localization of selected synaptic proteins, are unaltered in the cerebellum of *Myo16* knockout mice.

### Myosin XVI Localizes to Purkinje Cell Dendritic Spines

**FIGURE 2:**
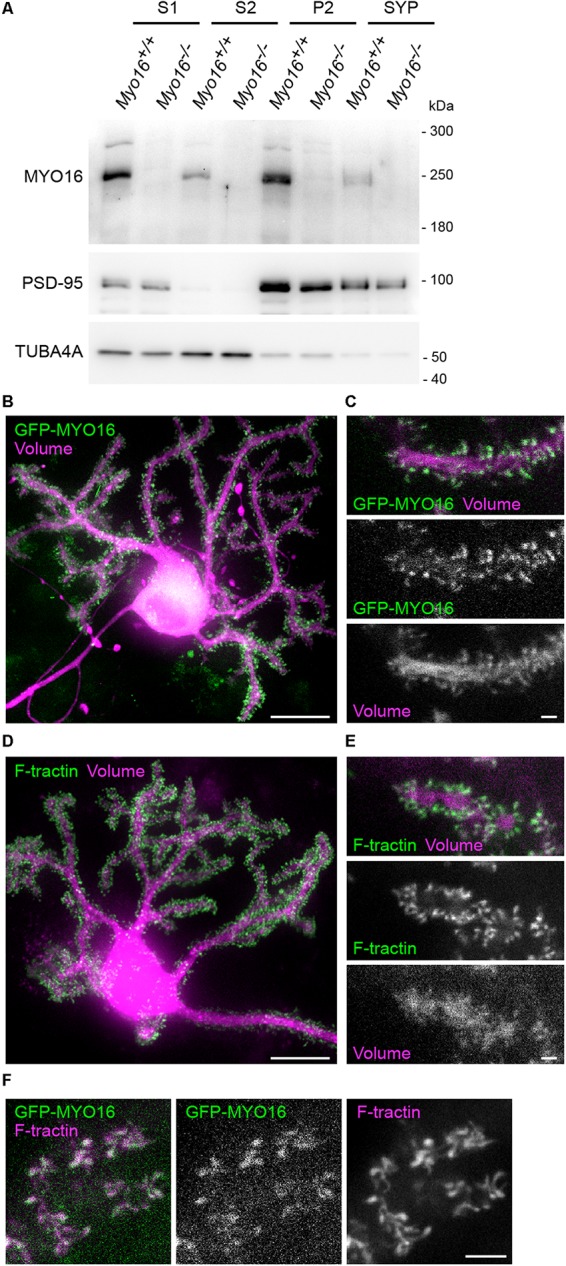
MYO16 localizes to the synaptosomal fraction and to Purkinje cell dendritic spines. **(A)** Cerebellar extracts of 3–4 week old *Myo16^+/+^* and *Myo16^*em*2^* knockout mice (*Myo16^–/–^*) were subjected to subcellular fractionation. S1, S2, P2, and SYP fractions were analyzed by Western blotting using antibodies against MYO16 (pre-adsorbed), PSD-95 (marker for membrane/synaptosomes), and alpha-Tubulin 4A (TUBA4A; marker for loss of cytosolic components). Note that specific MYO16 signal is present in P2 and SYP fractions (*n* = 3 experiments). **(B)** GFP-tagged myosin XVI heavy chain accumulates within the dendritic spines of Purkinje (cells (PCs). Cultured live PC at 14 DIV and co-transfected with *L7/Pcp-2* promoter plasmids for PC-specific expression of mGFP-MYO16 (green) and FusionRed (volume marker; magenta). Shown is a maximum projection of a *Z*-stack of images recorded using spinning disk confocal microscopy. Scale bar, 20 μm. **(C)** Dendrite branch of a live PC at 14 DIV transfected as in **(B)**. Shown are images of a single *Z*-plane recorded using spinning disk confocal microscopy. Images correspond to a frame of a time-lapse movie (Supplementary Movie S1). Scale bar, 2 μm. **(D)** Cultured live PC at 15 DIV and co-transfected with *L7/Pcp-2* promoter plasmids encoding the live cell F-actin marker F-tractin (green) and FusionRed (volume marker; magenta). Shown is a maximum projection of a *Z*-stack of images recorded using spinning disk confocal microscopy. Scale bar, 20 μm. **(E)** Dendrite branch of a live PC at 15 DIV transfected as in **(D)**. Shown are images of a single *Z*-plane recorded using spinning disk confocal microscopy. Images correspond to a frame of a time-lapse movie (Supplementary Movie S2). Scale bar, 2 μm. **(F)** Dendritic spines of a live PC at 15 DIV co-transfected with plasmids encoding mGFP-MYO16 (green) and F-tractin (magenta). Shown are spinning disk confocal images of a single *Z*-plane that correspond to a frame in a time-lapse movie (Supplementary Movie S3). Scale bar, 5 μm.)

In order to obtain insight into the subcellular locations at which endogenous myosin XVI might act, we determined its distribution using fractionation of cerebellar extracts (Figure 2A). MYO16 was detected in crude extracts (S1), but also in the membrane-enriched and synaptosomal fractions (P2, SYP), suggesting the possibility that the myosin localizes at or close to synapses. Successful enrichment of membranes and synaptosomes in P2 and SYP fractions was verified via PSD-95 enrichment and loss of tubulin subunit alpha-Tubulin-4A (Figure 2A). Since MYO16 is present in cerebellar PCs (Patel et al., 2001; Cameron et al., 2007), we further examined its subcellular location in this cell type. We made use of heterogeneous cerebellar cultures transfected with PC-specific expression plasmids as described (Wagner et al., 2011b) (see section “Materials and Methods”). Observation of live PCs expressing a red-fluorescent cell volume marker and GFP-tagged myosin XVI heavy chain (GFP-MYO16) showed that the myosin accumulates in essentially all dendritic spines (Figures 2B,C). Time-lapse movies of PC dendrites revealed that the GFP-MYO16 clusters change their shape over time (Supplementary Movie S1). These dynamic morphology changes were reminiscent of F-actin in the dendritic spines of live PCs (Figures 2D,E; see also Supplementary Movie S2), as visualized via F-tractin (Johnson and Schell, 2009). Indeed, GFP-MYO16 and F-tractin co-localized in spines of live PCs (Figure 2F; see also Supplementary Movie S3). Thus, the myosin XVI heavy chain localizes to the postsynaptic, F-actin rich spines of cerebellar PCs.

### Purkinje Cell Spine F-Actin Turnover Is Faster in the Absence of Myosin XVI

**FIGURE 3:**
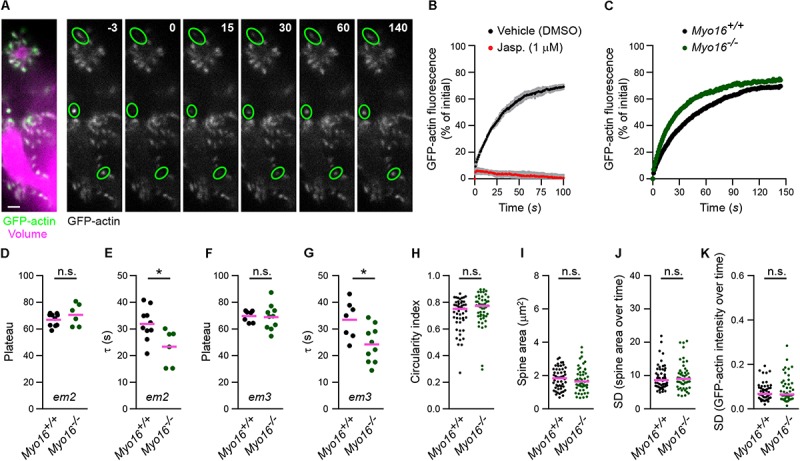
*Myo16* knockout leads to accelerated F-actin turnover in Purkinje cell dendritic spines. **(A)** FRAP analysis of GFP-actin in PC spines. Left, Dendrite branches of a live PC at 14 DIV, co-transfected with *L7/Pcp-2* promoter plasmids encoding GFP-actin (green) and volume marker FusionRed (magenta). Shown is an image recorded using spinning disk confocal microscopy. Scale bar, 2 μm. Right, Example of a FRAP experiment. Images of GFP-actin taken before and after bleaching are shown, time is indicated (seconds). Green ovals highlight bleached spines. Images correspond to frames of a time-lapse movie (Supplementary Movie S4). **(B)** FRAP analysis of GFP-actin in spines of PCs treated with 1 μM jasplakinolide (Jasp.; red) or with vehicle (0.1% [v/v] DMSO; black). Cells were co-transfected with *L7/Pcp-2* promoter plasmids encoding GFP-actin and volume marker as in **(A)**. Graph depicts recovery of GFP-actin fluorescence intensity in spines over time (s) relative to the bleached fluorescence intensity. Data points represent mean (*n* = 3 independent experiments per condition) ± SD (gray). **(C)** FRAP analysis of GFP-actin in spines of *Myo16^*em*2^* knockout PCs (*Myo16^–/–^*) and *Myo16^+/+^* littermate PCs co-transfected as in **(A)**. Graph depicts recovery of GFP-actin fluorescence intensity in spines, data points represent the mean of a representative experiment (see section “Materials and Methods”). **(D)** GFP-actin FRAP recovery plateau in spines of *Myo16^*em*2^* knockout PCs (*Myo16^–/–^*) and *Myo16^+/+^* littermate PCs. Data show plateau values obtained as described in section “Materials and Methods” from independent experiments (*n* = 6–10; magenta line indicates mean); *p* value determined using Student’s *t*-test. **(E)** GFP-actin FRAP recovery time constant (τ) in spines of *Myo16^*em*2^* knockout PCs (*Myo16^–/–^*) and *Myo16^+/+^* littermate PCs. Data are τ values obtained as described in section “Materials and Methods” from independent experiments (*n* = 6–10; magenta line indicates mean); *p* value determined using Student’s *t*-test. **(F,G)** As in **(D,E)**, but using *Myo16^*em*3^* knockout PCs (*Myo16^–/–^*) and *Myo16^+/+^* littermate PCs (*n* = 7–10). **(H)** Circularity index of spines of *Myo16^*em*2^* knockout PCs (*Myo16^–/–^*) and *Myo16^+/+^* littermate PCs expressing GFP-actin and volume marker. Value of 1.0 corresponds to perfectly circular shape, lower values indicate elongated shape. Data points represent single spines, magenta line indicates median; *p* value determined using Mann–Whitney test. **(I)** Apparent area covered by single spines of *Myo16^*em*2^* knockout PCs (*Myo16^–/–^*) and *Myo16^+/+^* littermate PCs expressing GFP-actin and volume marker. Data points represent single spines, magenta line indicates median; *p* value determined using Student’s *t*-test. **(J)** Spine area changes over time of *Myo16^*em*2^* knockout PCs (*Myo16^–/–^*) and *Myo16^+/+^* littermate PCs expressing GFP-actin and volume marker. Data points represent standard deviation (SD) of the relative area change of single spines over 150 s, magenta line indicates median; *p* value determined using Mann–Whitney test. **(K)** Change of GFP-actin fluorescence intensity over time in spines of *Myo16^*em*2^* knockout PCs (*Myo16^–/–^*) and *Myo16^+/+^* littermate PCs expressing GFP-actin and volume marker. Data points represent SD of the relative fluorescence change of single spines over 150 s, magenta line indicates median. For reason of comparability with the other figures, a single data point of *Myo16^–/–^* lying above the *Y*-axis limit is not shown; *p* value determined using Mann–Whitney test. ^*^p < 0.05; ^∗∗^*p* < 0.01; ^∗∗∗^*p* < 0.001; ^****^p < 0.0001; n.s., not significant.

Given the localization of MYO16 to PC spines, combined with the known ability of the myosin to bind the WRC (Yokoyama et al., 2011), we wondered whether the myosin is involved in regulating actin polymerization in spines. In order to monitor F-actin turnover in dendritic spines of *Myo16^–/–^* PCs, the beta isoform of actin was tagged with monomeric GFP (GFP-actin) and expressed in cultured PCs. GFP-actin is widely used to monitor F-actin dynamics and turnover in hippocampal and cortical spines (e.g., Star et al., 2002; Okamoto et al., 2007; Hotulainen et al., 2009; Rex et al., 2010; Koskinen et al., 2012, 2014; Kim et al., 2013; Chazeau et al., 2015; Chen et al., 2015). As anticipated, GFP-actin accumulated in the spines of live PCs (Figure 3A). To assess the turnover of GFP-actin in single PC spines, we used FRAP (Figure 3A; see also Supplementary Movie S4). To verify that FRAP of GFP-actin reveals the turnover of actin filaments in PC spines (as opposed to diffusion of GFP-actin monomers into spines), cells were treated with the F-actin stabilizing drug jasplakinolide (1 μM) (Bubb et al., 1994; Cramer, 1999). As expected, while GFP-actin fluorescence recovered in spines of vehicle-treated cells following bleaching, recovery was almost entirely blocked upon jasplakinolide treatment (Figure 3B). This confirms that the observed fluorescence recovery reflects formation of new F-actin in PC spines.

To determine whether myosin XVI is required for F-actin turnover, we performed FRAP analysis of GFP-actin in spines of *Myo16^–/–^* PCs (*Myo16^*em*2^* allele; Figure 3C). The plateau of fluorescence recovery indicates the fraction of F-actin that undergoes turnover (referred to as the mobile F-actin pool), while the recovery time constant (τ) is a measure of F-actin turnover rate in spines (Star et al., 2002). Our analyses did not reveal a significant difference regarding the mobile pool of spine F-actin when comparing *Myo16^–/–^* to wild-type littermate PCs (71 vs. 67%, respectively; Figure 3D). However, F-actin turnover rate was significantly faster (i.e., τ was smaller) in *Myo16^–/–^* PC spines compared to control (τ = 23 s vs. τ = 32 s, respectively; Figure 3E). We independently confirmed these results using PCs from the knockout mouse line carrying the *Myo16^*em*3^* allele (Figures 3F,G). Therefore, F-actin turnover in PC spines is accelerated upon *Myo16* knockout.

Since the actin cytoskeleton is a crucial determinant of spine shape (Hotulainen and Hoogenraad, 2010), we also monitored whether overall morphology and dynamics of spines are changed in *Myo16^–/–^* PCs. Using images of unbleached spines recorded during the GFP-actin FRAP experiments, a spine circularity index was determined as a measure for spine shape (Figure 3H). Moreover, we monitored spine area (an indirect measure of spine size; Figure 3I), relative spine area size changes over 2.5 min (Figure 3J), and relative changes of actin fluorescence intensity in spines over time (Figure 3K). None of these parameters were significantly different in *Myo16^–/–^* PCs when compared to wild-type. Thus, overall morphology and dynamics of PC spines appear to be normal in the absence of myosin XVI.

### Myosin XVI Acts Within Purkinje Cells to Regulate Spine F-Actin Turnover

**FIGURE 4:**
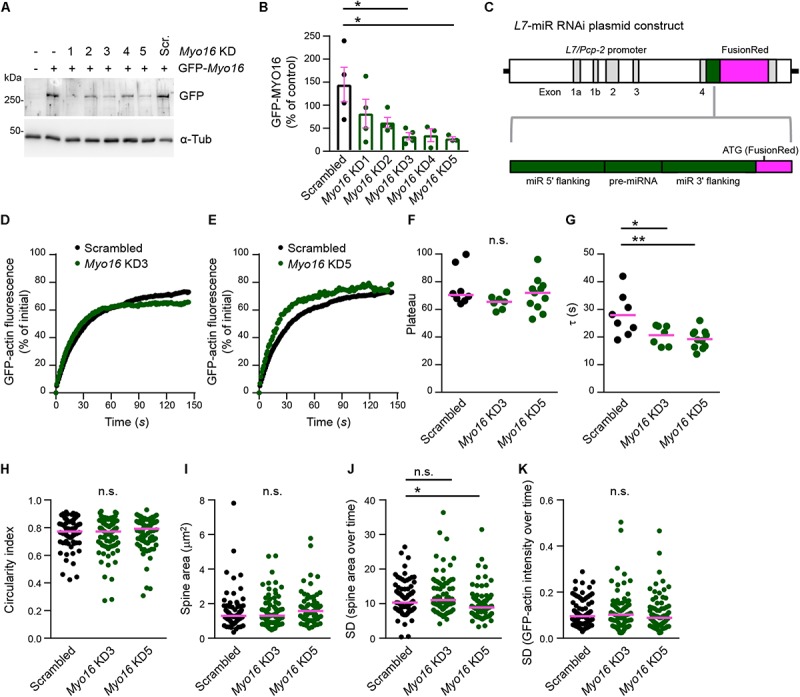
Purkinje cell-specific *Myo16* knockdown leads to accelerated F-actin turnover in dendritic spines. **(A,B)** Identification of *Myo16* miR RNAi knockdown constructs. **(A)** Western blot analysis of HEK293 cells co-transfected with a plasmid encoding mGFP-tagged mouse MYO16 and pcDNA^TM^6.2-GW/EmGFP-miR plasmids carrying the indicated knockdown sequences (*Myo16* KD1–KD5) or scrambled control (Scr.). For control, untransfected cells (first lane) and cells transfected only with plasmid encoding mGFP-*Myo16* were used. Antibodies against GFP and α-Tubulin (α-Tub; loading control) were used. **(B)** Quantification of GFP-MYO16 protein amount upon co-transfection with *Myo16* knockdown constructs KD1–KD5 or scrambled control. Graph depicts GFP-MYO16 signals normalized to tubulin signals and expressed as percentage of control (i.e., cells transfected with GFP-MYO16 plasmid only). Bars indicate mean values (*n* = 3–4; data points represent experiments) ± SEM (magenta); *p* values determined using Kruskal–Wallis test (*p* = 0.0321) followed by Dunn’s multiple comparisons test. **(C)** Schematic representation of plasmids for expressing *Myo16* KD3, *Myo16* KD5 or scrambled sequence (*pre-miRNA*) and flanking miR sequences (green) together with FusionRed as a reporter for RNAi expression (magenta) under control of the PC-specific *L7/Pcp-2* promoter. **(D,E)** FRAP analysis of GFP-actin in spines of wild-type PCs transfected with *L7/Pcp-2* promoter plasmids carrying *Myo16* KD3, *Myo16* KD5, or scrambled sequence and FusionRed (reporter for knockdown construct expression). Cells were co-transfected with a plasmid encoding GFP-actin. Graphs depict recovery of GFP-actin fluorescence intensity in spines, data points represent the mean of a representative experiment. For clarity, recovery curves of *Myo16* KD3 **(D)** and *Myo16* KD5 **(E)** are compared to the same scrambled control but shown in separate graphs. **(F)** GFP-actin FRAP recovery plateau in spines of PCs transfected as described in **(D,E)**. Data are plateau values obtained from independent experiments (*n* = 7–11; magenta line indicates median); *p* value determined using Kruskal–Wallis test. **(G)** GFP-actin FRAP recovery time constant (τ) in spines of PCs transfected as described in **(D,E)**. Data are τ values from independent experiments (*n* = 7–11; magenta line indicates mean); *p* values determined using one-way ANOVA (*p* = 0.0038) followed by Tukey’s multiple comparisons test. **(H)** Circularity index of spines of PCs transfected as described in **(D,E)**. Data points represent single spines, magenta line indicates median; *p* value determined using Kruskal–Wallis test. **(I)** Apparent area covered by single spines of PCs transfected as described in **(D,E)**. Data points represent single spines, magenta line indicates median; *p* value determined using Kruskal–Wallis test. **(J)** Spine area changes over time of PCs transfected as described in **(D,E)**. Data points represent SD of the relative area change of single spines over 150 s, magenta line indicates median; *p* values determined using Kruskal–Wallis test (*p* = 0.0008) followed by Dunn’s multiple comparisons test. **(K)** Change of GFP-actin fluorescence intensity over time in spines of PCs transfected as described in **(D,E)**. Data points represent SD of the relative fluorescence change of single spines monitored over 150 s, magenta line indicates median. For reasons of comparability with the other figures, a single data point of *Myo16* KD5 lying above the *Y*-axis limit is not shown; *p* value determined using Kruskal–Wallis test. ^*^p < 0.05; ^∗∗^*p* < 0.01; ^∗∗∗^*p* < 0.001; ^****^p < 0.0001; n.s., not significant.

Myosin XVI might affect F-actin turnover directly via acting in PC spines. Alternatively, altered F-actin dynamics in *Myo16^–/–^* PCs might be of non-cell autonomous origin such as altered presynaptic input from granule cells. Thus, to determine whether the myosin is required within PCs, we performed PC-specific *Myo16* knockdown. First, we identified RNAi sequences that, when embedded in a miR backbone, knockdown *Myo16* expression (Figure 4A). Compared to control levels, the independent knockdown sequences *Myo16* KD3 and *Myo16* KD5 significantly reduced GFP-MYO16 expression to 33% and 28%, respectively (Figure 4B). Following a previously used strategy (Alexander and Hammer, 2016), these knockdown sequences as well as a reporter (FusionRed) were placed under control of the *L7/Pcp-2* promoter (Figure 4C) which drives PC-specific expression in cerebellar cultures (Wagner et al., 2011b). Expression of the *Myo16* KD3, *Myo16* KD5 or scrambled miR knockdown constructs in the analyzed PCs was verified via monitoring FusionRed.

Fluorescence recovery after photobleaching analysis of GFP-actin showed that, similar to the situation in *Myo16^–/–^* PCs, the two independent PC-specific *Myo16* knockdown constructs both caused a significantly faster turnover of F-actin in spines compared to scrambled control (*Myo16* KD3, τ = 21 s; *Myo16* KD5, τ = 19 s; Scrambled, τ = 28 s), while the mobile pool of F-actin remained unchanged (Figures 4D–G). Moreover, as in case of *Myo16^–/–^* PCs, no consistent changes in terms of overall morphology and dynamics of spines were observed upon *Myo16* knockdown (Figures 4H–K). Therefore, both global *Myo16* knockout and PC-specific *Myo16* knockdown lead to an identical phenotype, i.e., accelerated F-actin turnover in PC-spines. Since GFP-MYO16 targets to PC spines (Figure 2), this indicates that the myosin functions at the postsynaptic side to attenuate spine F-actin turnover.

### Faster F-Actin Turnover in Purkinje Cell Spines Upon WRC Inhibition

**FIGURE 5:**
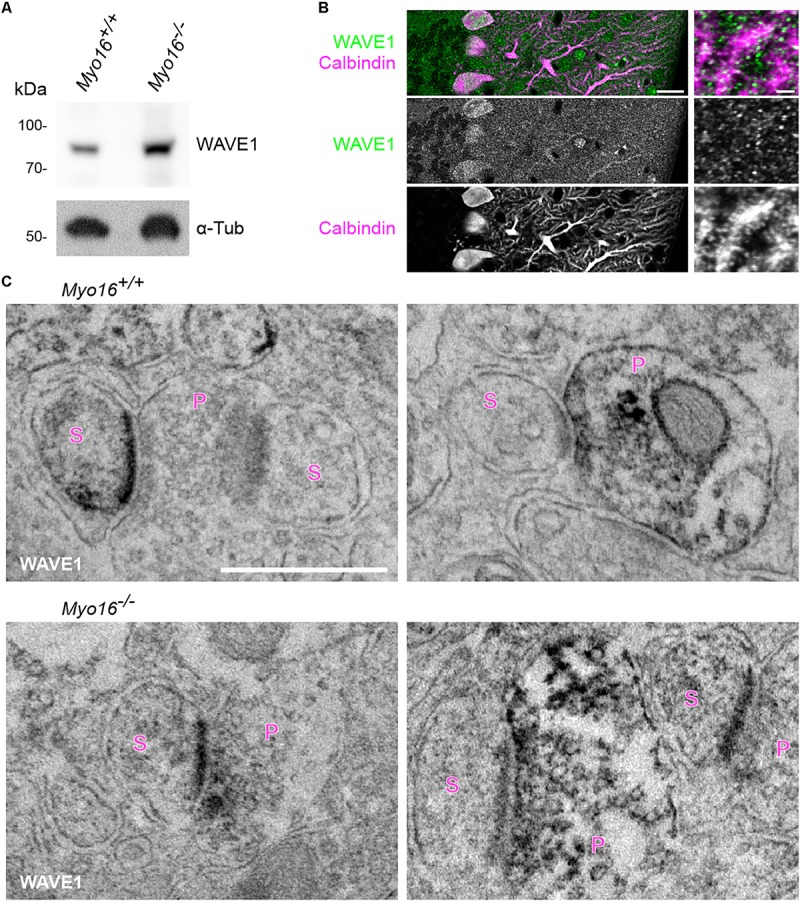
WAVE1 localizes to Purkinje cell spines and to presynaptic terminals in the cerebellar molecular layer. **(A)** Western blot analysis of cerebellar extracts from 11 day old wild-type (*Myo16^+/+^*) and *Myo16^*em*3^* knockout (*Myo16^–/–^*) mice. Antibodies against WAVE1 and α-Tubulin (α-Tub; loading control) were used. **(B)** WAVE1 partially co-localizes with PC spines. Confocal images of cerebellar sections from adult wild type mice immuno-fluorescently labeled with antibodies against WAVE1 and Calbindin-D-28K. Right panels are zoom-in images depicting the molecular layer. Scale bars, 20 μm (right panels); 2 μm (left panels). **(C)** Immuno-electron microscopy of cerebellar sections from adult wild-type mice (*Myo16^+/+^*; upper panels) and *Myo16^*em*3^* knockout mice (*Myo16^–/–^*; lower panels) using WAVE1 antibody and DAB labeling. Black precipitate indicates presence of WAVE1. Left panels depict examples of PC spines positive for WAVE1, right panels are examples of presynaptic terminals positive for WAVE1. S, spine; P, presynaptic bouton. Scale bar, 500 nm.

The WRC is an activator of Arp2/3-mediated F-actin polymerization (Bisi et al., 2013) and a well-established interaction partner of myosin XVI (Yokoyama et al., 2011). Therefore, we asked whether also the WRC is important for actin cytoskeleton dynamics in dendritic spines of PCs. We first determined whether the WRC-subunit WAVE1 localizes to PC spines *in situ*. We employed an anti-WAVE1 antibody that detects a band of expected size in cerebellar extracts (Figure 5A). Immuno-fluorescence labeling of wild-type cerebellum confirmed (Soderling et al., 2003) the presence of WAVE1 in PCs and revealed partial co-localization with PC spines (Figure 5B). Immuno-electron microscopy showed that WAVE1 is present at pre- and postsynaptic sites within the cerebellar molecular layer (Figure 5C, *Myo16^+/+^* panels). In spines, predominantly the postsynaptic density was labeled (Figure 5C, left *Myo16^+/+^* panel). In cerebellum of *Myo16^–/–^* mice, no obvious difference in WAVE1 labelling was observed (Figure 5C, *Myo16^–/–^* panels). Thus, WAVE1 is present in PC spines, and myosin XVI is not essential for WAVE1 targeting to spines.

**FIGURE 6:**
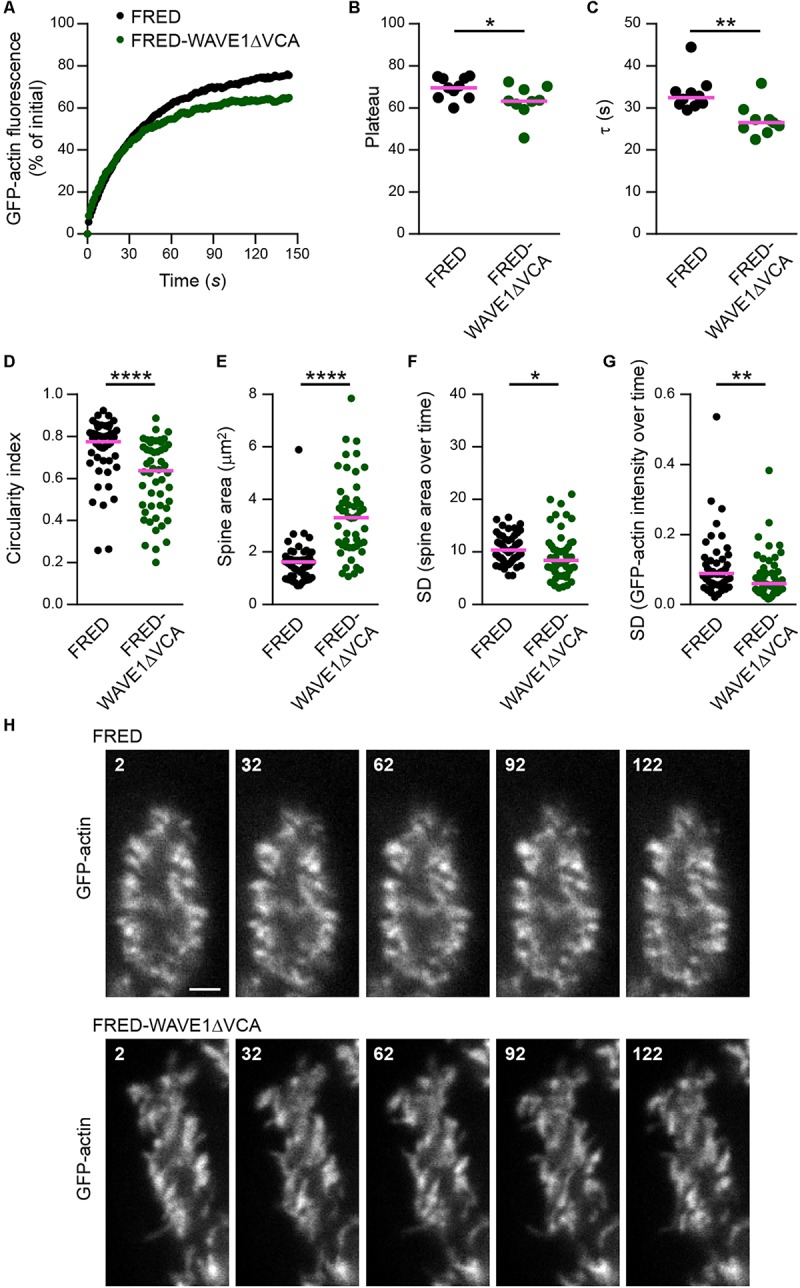
WAVE1ΔVCA leads to accelerated F-actin turnover and a decreased dynamic F-actin pool in Purkinje cell dendritic spines. **(A)** FRAP analysis of GFP-actin in spines of wild-type PCs transfected with *L7/Pcp-2* promoter plasmids encoding either FusionRed-tagged WAVE1 lacking the VCA domain (FRED-WAVE1ΔVCA) or FusionRed alone (FRED). Cells were co-transfected with a plasmid encoding GFP-actin. Graph depicts recovery of GFP-actin fluorescence intensity in spines, data points represent the mean of a representative experiment. **(B)** GFP-actin FRAP recovery plateau in spines of PCs transfected ( as described in **(A)**. Data represent plateau values obtained from independent experiments (*n* = 9–10; magenta line indicates mean); *p* value determined using Student’s *t*-test. **(C)** GFP-actin FRAP recovery time constant (τ) in spines of PCs transfected as described in **(A)**. Data represent τ values obtained from independent experiments (*n* = 9–10; magenta line indicates median); *p* value determined using Mann–Whitney test. **(D)** Circularity index of spines of PCs transfected as in **(A)**. Data points represent single spines, magenta line indicates median; *p* value determined using Mann–Whitney test. **(E)** Apparent area covered by single spines of spines of PCs transfected as in **(A)**. Data points represent single spines, magenta line indicates median; *p* value determined using Mann–Whitney test. **(F)** Spine area changes over time of PCs transfected as described in **(A)**. Data points represent SD of the relative area change of single spines over 150 s, magenta line indicates median; *p* value determined using Mann–Whitney test. **(G)** Change of GFP-actin fluorescence intensity over time in spines of PCs transfected as described in **(A)**. Data points represents SD of the relative fluorescence change of single spines over 150 s, magenta line indicates median; *p* value determined using Mann–Whitney test. **(H)** Examples of spines of PCs transfected as described in **(A)**. GFP-actin was visualized by spinning disk confocal microscopy (see also Supplementary Movies S5, S6). Time is indicated in seconds. Scale bar, 2 μm. ^*^p < 0.05; ^∗∗^*p* < 0.01; ^∗∗∗^*p* < 0.001; ^****^p < 0.0001; n.s., not significant.)

To interfere with WRC function, we made use of a well-characterized dominant-negative version of WAVE1 that lacks the C-terminal VCA domain (WAVE1ΔVCA) and thus is unable to activate Arp2/3 (Miki et al., 1998; Kim H.J. et al., 2006; Pils et al., 2012). To selectively target the PCs in heterogeneous cerebellar culture, WAVE1ΔVCA was expressed under control of the *L7/Pcp-2* promoter. GFP-actin FRAP analysis revealed that WAVE1ΔVCA leads to a significantly smaller mobile F-actin pool in PC spines (FRED-WAVE1ΔVCA: plateau at 63%; FRED control: plateau at 70%; Figures 6A,B). Moreover, the mobile pool displayed faster turnover (FRED-WAVE1ΔVCA, τ = 27 s; FRED control, τ = 32 s; Figure 6C). Therefore, interference with the WRC within PCs phenocopies both *Myo16* knockout and knockdown in accelerating F-actin turnover in spines.

Analyses of PCs expressing GFP-actin showed furthermore that WAVE1ΔVCA leads to changes in spine morphology. Compared to control, spines of PCs that express WAVE1ΔVCA were more elongated and larger (Figures 6D,E,H; see also Supplementary Movies S5, S6). In addition, WAVE1ΔVCA attenuated size changes and actin intensity fluctuation in PC spines over time (Figures 6F,G). This indicates that the WRC promotes circular spine shape and favors spine dynamics in PCs.

### Arp2/3 Inhibition, but Not Formin Inhibition, Accelerates F-Actin Turnover in PC Spines

**FIGURE 7:**
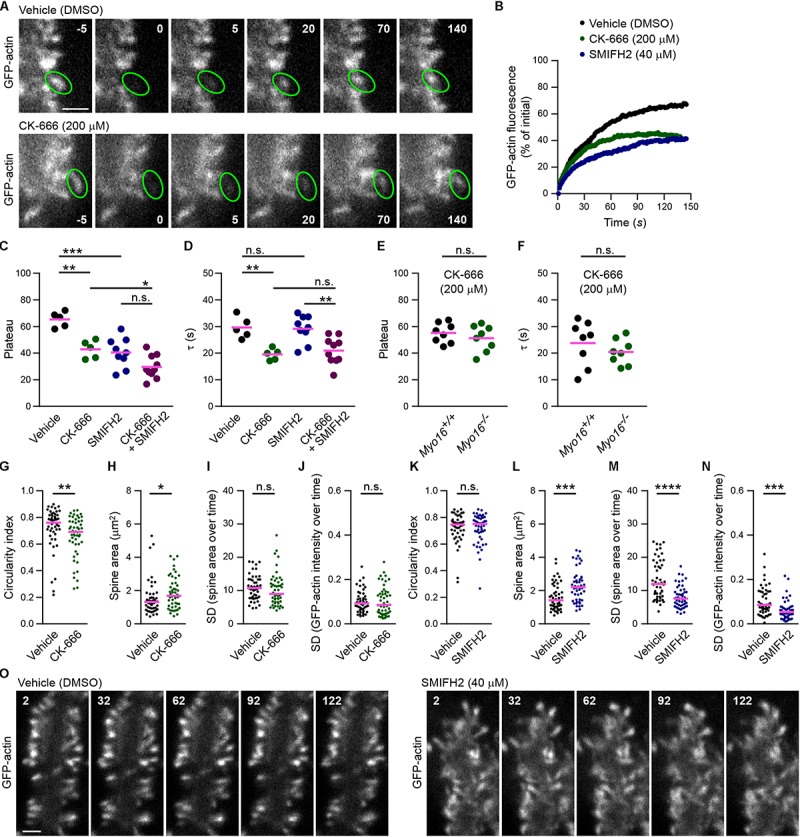
Arp2/3 inhibition, but not formin inhibition, accelerates F-actin turnover in PC spines. **(A)** Examples of GFP-actin FRAP in PC spines in the absence or presence of Arp2/3 inhibitor CK-666. Wild-type PCs were co-transfected with *L7/Pcp-2* promoter plasmids encoding GFP-actin and volume marker FusionRed. Shown are images depicting GFP-actin and recorded using spinning disk confocal microscopy. Upper row, vehicle-treated control (0.4% [v/v] DMSO). Lower row, treated with CK-666 (final concentration, 200 μM). Time before and after bleaching is indicated (seconds). Green ovals highlight bleached spines. Scale bar, 2 μm. **(B)** FRAP analysis of GFP-actin in spines of PCs co-transfected as in **(A)** and treated with CK-666 (200 μM; green), formin inhibitor SMIFH2 (40 μM; blue), or vehicle (black). Graph depicts recovery of GFP-actin fluorescence intensity in spines, data points represent the mean of a representative experiment. **(C)** GFP-actin FRAP recovery plateau in spines of PCs transfected as in **(A)** and treated with CK-666, SMIFH2, CK-666 + SMIFH2, or vehicle. Data are plateau values obtained from independent experiments (*n* = 5–10; magenta line indicates mean); *p* values determined using one-way ANOVA (*p* < 0.0001) followed by Sidak’s multiple comparisons test. **(D)** GFP-actin FRAP recovery time constant (τ) in spines of PCs transfected as in **(A)** and treated with CK-666, SMIFH2, CK-666 + SMIFH2, or vehicle. Data are τ values obtained from independent experiments (*n* = 5–10; magenta line indicates mean); *p* values determined using one-way ANOVA (*p* = 0.0003) followed by Sidak’s multiple comparisons test. **(E)** GFP-actin FRAP recovery plateau in spines of *Myo16^*em*2^* knockout PCs (*Myo16^–/–^*) and *Myo16^+/+^* littermate PCs transfected as in **(A)** and treated with CK-666. Data represent plateau values obtained from independent experiments (*n* = 8; magenta line indicates mean); *p* value determined using Student’s *t*-test. **(F)** GFP-actin FRAP recovery time constant (τ) in spines of *Myo16^*em*2^* knockout PCs (*Myo16^–/–^*) and *Myo16^+/+^* littermate PCs transfected as in **(A)** and treated with CK-666. Data represent plateau values obtained from independent experiments (*n* = 8; magenta line indicates mean); *p* value determined using Student’s *t*-test. **(G)** Circularity index of spines of PCs treated with CK-666 or vehicle and transfected as in **(A)**. Data points (represent single spines, magenta line indicates median; *p* value determined using Mann–Whitney test. **(H)** Apparent area covered by single spines of PCs treated with CK-666 or vehicle and transfected as in **(A)**. Data points represents single spines, magenta line indicates median; *p* value determined using Mann–Whitney test. **(I)** Spine area changes over time of PCs treated with CK-666 or vehicle and transfected as in **(A)**. Data points represent SD of the relative area change of single spines monitored over 150 s, magenta line indicates median; *p* value determined using Mann–Whitney test. **(J)** Change of GFP-actin fluorescence intensity over time in spines of PCs treated with CK-666 or vehicle and transfected as in **(A)**. Data points represent the SD of the relative fluorescence change of single spines monitored over 150 s, magenta line indicates median; *p* value determined using Mann–Whitney test. **(K)** Circularity index of spines of PCs transfected as in **(A)** and treated with SMIFH2 (blue; final concentration, 40 μM) or with vehicle (0.2% [v/v] DMSO; black). Data points represent single spines, magenta line indicates median; *p* value determined using Mann–Whitney test. **(L)** Apparent area covered by single spines of PCs treated with SMIFH2 or vehicle and transfected as in **(A)**. Data points represent single spines, magenta line indicates median; *p* value determined using Mann–Whitney test. **(M)** Spine area changes over time of PCs treated with SMIFH2 or vehicle and transfected as in **(A)**. Data points represent SD of the relative area change of single spines monitored over 150 s, magenta line indicates median; *p* value determined using Mann–Whitney test. **(N)** Change of GFP-actin fluorescence intensity over time in spines of PCs treated with SMIFH2 or vehicle and transfected as in **(A)**. Data point represent SD of the relative fluorescence change of single spines monitored over 150 s, magenta line indicates median; *p* value determined using Mann–Whitney test. **(O)** Examples of spines of PCs treated with SMIFH2 or vehicle and transfected as described in **(A)**. GFP-actin was visualized by spinning disk confocal microscopy. Time is indicated in seconds. Scale bar, 2 μm. ^*^p < 0.05; ^∗∗^*p* < 0.01; ^∗∗∗^*p* < 0.001; ^****^p < 0.0001; n.s., not significant.)

Since Arp2/3 is the downstream target of the WRC (Takenawa and Suetsugu, 2007; Bisi et al., 2013), reduced Arp2/3 activation might be the cause of accelerated F-actin turnover upon WRC inhibition (Figure 6C). To determine whether Arp2/3 plays a role for F-actin turnover in PC spines, cells were acutely exposed to CK-666, a well-established small molecule inhibitor of Arp2/3 (Nolen et al., 2009), before performing FRAP analysis of GFP-actin (Figure 7A). Similar as for WRC inhibition, spines of PCs treated with 200 μM CK-666 displayed a significantly smaller mobile F-actin pool (CK-666, plateau at 43%; vehicle control, plateau at 65%; Figures 7B,C). Moreover, like when interfering with the WRC or with myosin XVI, turnover of the mobile F-actin pool was significantly faster upon CK-666 treatment (CK-666, τ = 20 s; vehicle control, τ = 30 s; Figure 7D). Together, our data indicate that the WRC-Arp2/3 pathway determines the relative level of mobile F-actin in PC spines and attenuates its turnover time.

We next tested whether also manipulation of actin polymerization factors unrelated to Arp2/3 results in accelerated F-actin turnover in PC spines. For this purpose, cells were acutely treated with SMIFH2 (40 μM), an inhibitor of formin-mediated actin filament nucleation and elongation (Rizvi et al., 2009). Similar to interference with WRC-Arp2/3, this led to a significantly smaller mobile F-actin pool compared to vehicle control (SMIFH2, plateau at 40%; Figures 7B,C). Thus, formin activity is required for F-actin dynamics in PC spines. Notably, however, the turnover time of the dynamic pool was not reduced compared to control (SMIFH2, τ = 29 s; Figure 7D). Moreover, the effects of CK-666 and SMIFH2 on mobile pool size of F-actin were additive, with both blockers leading to a significantly reduced plateau compared to CK-666 alone (CK-666+SMIFH2, plateau at 30%; Figure 7C). Therefore, WRC-Arp2/3 and formins appear to act in parallel pathways influencing F-actin dynamics in PC spines, with WRC-Arp2/3 – but not formins – attenuating F-actin turnover rate.

Since myosin XVI attenuates F-actin turnover rate in PC spines (Figures 3, 4) and binds to the WRC (Yokoyama et al., 2011), the myosin might act through the WRC-Arp2/3 pathway. If this is the case, *Myo16* knockout may not have an additive effect on actin dynamics upon Arp2/3 inhibition. Indeed, neither the mobile pool of spine F-actin nor its turnover rate was significantly different in spines of CK-666-treated *Myo16^–/–^* PCs when compared to CK-666-treated littermate, wild-type PCs (Figures 7E,F). This argues for myosin XVI regulating F-actin turnover rate in PC spines via Arp2/3.

Finally, we examined whether acute exposure to the Arp2/3 or formin inhibitors changes overall spine morphology or dynamics (Figures 7G–O). Similar to WRC inhibition (Figure 6D) or long-term (24 h) treatment with CK-666 (Kawabata Galbraith et al., 2018), short-term Arp2/3 inhibition led to a significantly more elongated spine shape (Figure 7G). Moreover, CK-666 treatment resulted in increased spine size (Figure 7H). Size changes of PC spines or actin intensity changes in spines over 2.5 min were not significantly affected by Arp2/3 inhibition (Figures 7I,J). This indicates that the WRC-Arp2/3 pathway promotes a circular spine shape in PCs, consistent with Arp2/3 driving the formation of a non-uniformly oriented, branched actin meshwork (Chazeau and Giannone, 2016). In contrast, acute formin inhibition affected PC spine morphology and dynamics differently (Figures 7K–O). Unlike CK-666, SMIFH2 did not lead to spine elongation (Figure 7K) but significantly reduced relative spine size changes and actin fluorescence intensity changes in spines over time (Figures 7M,N). Similar to CK-666 treatment, PC spines adopted a larger area upon exposure to SMIFH2 (Figure 7L). Therefore, unlike Arp2/3, formin activity is needed to promote fluctuations of PC spine size and actin content.

### Altered Synaptic Structure and Transmission in the Cerebellum of *Myo16* Knockout Mice

**FIGURE 8:**
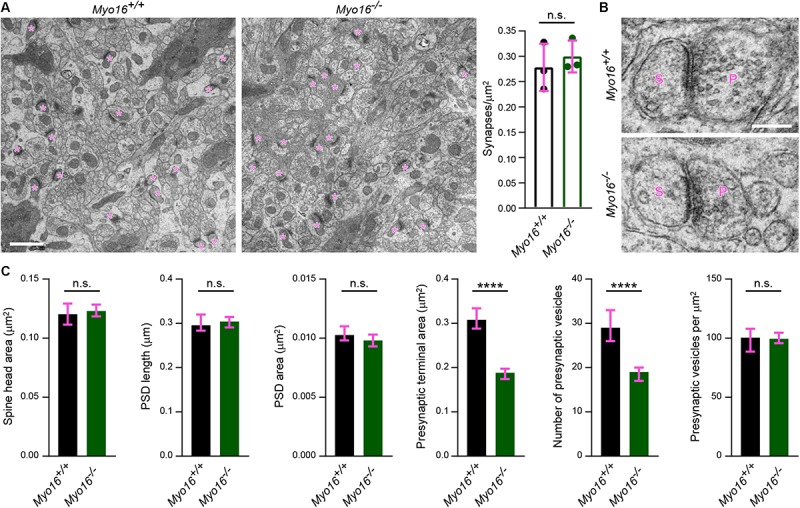
Ultrastructural analysis reveals altered presynaptic organization in the cerebellar molecular layer of *Myo16^–/–^* knockout mice. **(A)** Electron microscopy images of the middle one third of the cerebellar molecular layer of adult male *Myo16^*em*3^* knockout (*Myo16^–/–^*) and *Myo16^+/+^* littermate control mice were surveyed for asymmetric synapses (indicated by magenta asterisks). Graph shows number of synapses per μm^2^; bars indicate mean ± SEM (magenta), *n* = 3 (single data points represent mice analyzed), no significant differences detected (n.s.; *p* value determined using Student’s *t*-test). Scale bar, 1 μm. **(B)** Representative electron microscopy images of cerebellar molecular layer synapses of adult male *Myo16^*em*3^* knockout (*Myo16^–/–^*) and *Myo16^+/+^* littermate mice. S, spine; P, presynaptic bouton. Scale bar, 250 nm. **(C)** Quantitative analysis of morphological parameters of cerebellar molecular layer synapses of *Myo16^–/–^* and *Myo16^+/+^* mice. Data are shown as median ± 95% confidence interval (magenta), *n* = 236 synapses (*Myo16^+/+^*) and 380 (*Myo16^–/–^*); *p* values determined using Mann–Whitney tests. ^*^p < 0.05; ^∗∗^*p* < 0.01; ^∗∗∗^*p* < 0.001; ^****^p < 0.0001; n.s., not significant.

Having established that myosin XVI regulates actin dynamics in cerebellar PCs, we examined in more detail whether synapses formed on PC spines are structurally altered in *Myo16^–/–^* mice *in situ*. First, using electron microscopy images of the middle third of the molecular layer, we quantified the number of asymmetric synapses per μm^2^ (Figure 8A). No difference in the density of these excitatory synapses that mainly represent PF-PC synapses was detected. Consistent with the finding that spine size of cultured *Myo16^–/–^* PCs is unaltered (Figure 3I), also spine head area of molecular layer synapses measured from electron microscopy images was unaltered (Figures 8B,C). Moreover, PSD length, PSD area and presynaptic vesicle density were unchanged at these synapses (Figures 8B,C). Strikingly, however, the area of presynaptic axon terminals and the absolute number of synaptic vesicles per terminal were dramatically reduced in the molecular layer of *Myo16^–/–^* cerebellum. Therefore, myosin XVI appears to be important for presynaptic ultrastructure and synaptic vesicle numbers at PF-PC synapses.

**FIGURE 9:**
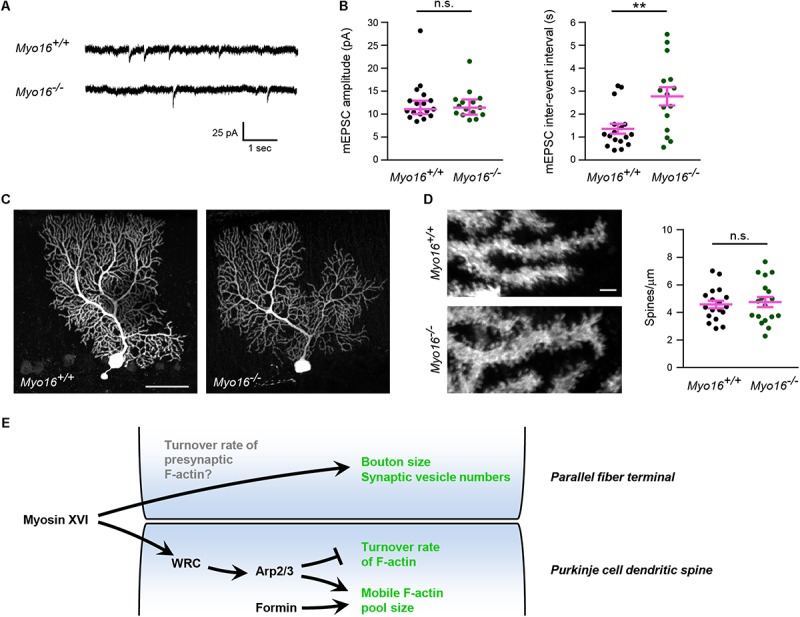
Increased inter-event interval of AMPA receptor-mediated mEPSCs in *Myo16* knockout PCs. **(A)** Example traces of mEPSC measurement from PCs in acute cerebellar slices of 3–4 week old *Myo16^*em*3^* knockout (*Myo16^–/–^*) and *Myo16^+/+^* littermate control mice. **(B)** Quantification of mEPSC amplitudes and inter-event intervals measured as in **(A)**. Data points represent analyzed PCs (*Myo16^+/+^*: *n* = 17 from seven mice; *Myo16^–/–^*: *n* = 15 from nine mice). mEPSC amplitude: median ± 95% confidence interval is indicated (magenta); *p* value determined using Mann–Whitney test. mEPSC inter-event interval: mean ± SEM is indicated (magenta); *p* value determined using Welch’s *t*-test. **(C)** PCs filled with biocytin during mEPSC measurement were stained and visualized using confocal microscopy. Images show *Z*-stack projections. Scale bar, 50 μm. **(D)** Higher magnification images of PC dendrites with spines obtained as in **(C)**. Scale bar, 2 μm. Graph depicts number of spines per μm dendrite. Data points represent analyzed PCs, mean ± SEM is indicated (magenta); *p* value determined using Student’s *t*-test. **(E)** Model of the role of myosin XVI at PF-PC synapses. In PC dendritic spines, myosin XVI is required for the attenuation of F-actin turnover. We propose that the myosin functions by promoting WRC activation which, in turn, activates Arp2/3-mediated branched F-actin formation. In addition to WRC and Arp2/3, also formins promote F-actin mobility in PC spines. However, interference with myosin XVI, WRC, and Arp2/3, but not with formins, accelerates F-actin turnover rate, consistent with formins acting in a separate pathway. At the presynaptic side, loss of myosin XVI leads to reduced numbers of synaptic vesicles and terminals of apparently reduced size. This may be a consequence (i) of a role of the myosin at the presynaptic side (e.g., in F-actin turnover) or (ii) of the postsynaptic changes in *Myo16^–/–^* PCs. ^*^p < 0.05; ^∗∗^*p* < 0.01; ^∗∗∗^*p* < 0.001; ^****^p < 0.0001; n.s., not significant.

Both altered postsynaptic actin dynamics and the ultrastructural abnormalities at presynaptic terminals observed in the absence of myosin XVI might affect the function of PF-PC synapses. To test whether *Myo16* knockout alters synaptic transmission onto PCs, we measured spontaneous AMPA receptor-mediated mEPSCs from PCs in acute cerebellar slices (Figure 9A). No difference in the peak amplitude of mEPSCs was observed when comparing *Myo16^–/–^* and wild-type littermate PCs (Figure 9B), suggesting that synaptic AMPA receptor content is unchanged. In contrast, the mEPSC inter-event interval was significantly longer in *Myo16^–/–^* PCs (Figure 9B). Such a reduced frequency of mEPSCs may arise from a reduced number of synapses formed on the PCs. However, no gross abnormalities in dendritic arborization of the analyzed PCs was observed (Figure 9C). Moreover, spine density on *Myo16^–/–^* PCs was unaltered (Figure 9D), and the density of excitatory molecular layer synapses was unchanged in *Myo16^–/–^* cerebellum (Figure 8A). Therefore, the increase in mEPSC inter-event interval provides further support for a reduced number of presynaptic vesicles at PC synapses in *Myo16* knockout cerebellum (Figures 8B,C).

### *Myo16* Knockout Mice Show Comparable Performance to Controls in Motor- and Social Interaction-Tests

**FIGURE 10:**
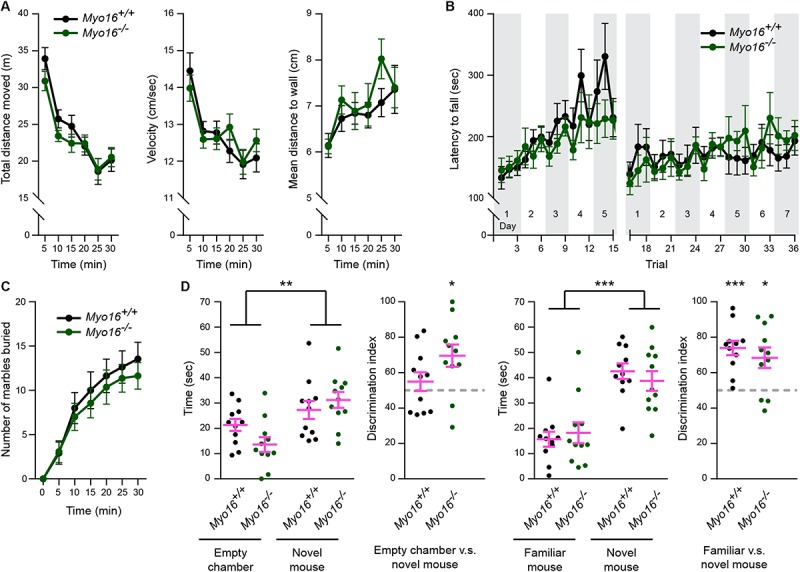
Comparable performance of *Myo16^*em*3^* knockout (*Myo16^–/–^*) and *Myo16^+/+^* littermate control mice in a behavior test survey. **(A)** In the open field test, both genotypes showed similar locomotor habituation to the paradigm as indicated by the decrease in distance moved (genotype: *p* = 0.42), decreased mean velocity (genotype: *p* = 0.88) and an increased mean distance to wall (less thigmotaxis) (genotype: *p* = 0.47) over a 30 min test duration. **(B)** Latency to fall from an accelerating rotarod revealed significant increase of duration over trials (*p* < 0.001) with no effect of genotype (*p* = 0.77). **(C)** Both genotypes show similar performance in marble burying (*p* = 0.61). **(D)** Time exploring an empty cup vs. a novel mouse, or a familiar vs. a novel mouse during the first 2 min of a social recognition test. Mice spent more exploration time with the novel mouse vs. the empty cup (*p* < 0.01, no effect of genotype: *p* = 0.17) and more time investigating the novel mouse vs. the familiar mouse (choice: *p* < 0.001, no effect of genotype: *p* = 0.82). The discrimination index showed a significant preference of *Myo16^–/–^* but not the *Myo16^+/+^* mice for the novel mouse over the empty chamber (*p* < 0.05; *p* = 0.372, respectively) and a significant preference of both genotypes for the novel over the familiar mouse (*p* < 0.05; *p* < 0.001, respectively). Values represent mean ± SEM (**A–D**: repeated measures ANOVA, Discrimination index: one-sample *t*-test, test value: 50). *Myo16^–/–^* and *Myo16^+/+^* mice: *n* = 11 each. ^*^p < 0.05; ^∗∗^*p* < 0.01; ^∗∗∗^*p* < 0.001; ^****^p < 0.0001; n.s., not significant.

Given the synaptic roles of myosin XVI identified so far (Figure 9E), we examined whether *Myo16^–/–^* mice display behavioral abnormalities. In the open field test (Figure 10A), *Myo16^–/–^* mice were indistinguishable from wild-type littermate controls in terms of locomotor activity (total distance moved, velocity) and regarding a measure of anxiety (mean distance to wall). Since we found that MYO16 is important for F-actin dynamics in cerebellar PCs and that synaptic organization in the cerebellum is altered in *Myo16^–/–^* mice, we next monitored the motor learning ability of the knockout mice. In the accelerating rotarod test, both *Myo16^–/–^* and wild-type control mice improved their performance over the first five test days, without differing significantly between genotypes (Figure 10B). Therefore, mice lacking myosin XVI perform similar to wild-type controls in this test for motor learning. Given the genetic association of *MYO16* and ASD, we also examined *Myo16^–/–^* mice for behaviors altered in mouse models of ASD. A marble burying test did not reveal differences in repetitive behavior of *Myo16^–/–^* mice compared to littermate controls (Figure 10C). Moreover, a social interaction test did not reveal deficits of *Myo16^–/–^* mice in their ability to discriminate between an empty chamber or a novel mouse, or between a familiar and a novel mouse (Figure 10D). Therefore, at least this limited set of behavioral assays did not reveal gross abnormalities in mouse behavior upon *Myo16* knockout.

## Discussion

Here, we define myosin XVI as a novel component of the regulatory machinery of actin turnover in dendritic spines of cerebellar PCs, and we uncover that myosin XVI is important for normal presynaptic organization in the cerebellum.

Our results shed light on the mechanisms that govern F-actin turnover in spines of PCs, a neuronal cell type implicated in motor learning, social behavior and ASD. We show that, similar to other neurons, the WRC-Arp2/3 pathway is crucial for the regulation of postsynaptic actin turnover in PCs. Interference with either WRC or Arp2/3 led to a reduced mobile pool and faster F-actin turnover in spines (FRAP- and PC spine measurement-results are summarized in Table 1). Notably, our observation that acute inhibition of Arp2/3 accelerates turnover rate in dendritic spines is consistent with the finding that CK-666 increases retrograde actin flow rates at the leading edge of neuronal growth cones (Yang et al., 2012). In comparison, diverse outcomes for postsynaptic F-actin turnover upon interfering with Arp2/3 or WRC have been reported previously for other neurons. For example, sparse *in vivo* knockout of the Arp2/3 subunit *ArpC3* in a subset of hippocampal neurons leads to a reduced mobile pool and slower recovery (Kim et al., 2013). Moreover, WAVE1 knockdown was found to reduce the mobile pool of F-actin in cortical spines (Njoo et al., 2015). In contrast, heterozygous knockout of the WRC subunit *Cyfip1* increases the mobile pool while leaving the recovery rate of spine F-actin unchanged (Pathania et al., 2014).

**TABLE 1:** Summary of GFP-actin FRAP and morphology/dynamics of Purkinje cell dendritic spines.

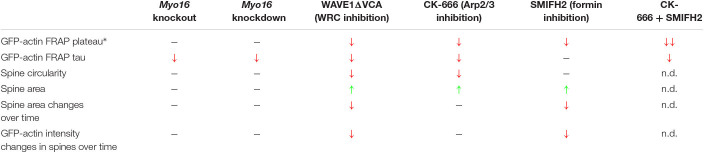

^*^Red arrows indicate significant decrease relative to control; green arrows indicate significant increase relative to control; black line indicates no change; n.d., not determined.

While Arp2/3 drives the formation of a non-uniformly oriented F-actin meshwork, formins promote the formation of linear actin filaments. We show that formins are also crucial for actin cytoskeleton dynamics in PC dendritic spines, as formin inhibition dramatically reduces the size of the mobile F-actin pool (Table 1). The effect of SMIFH2 on mobile pool size being additive to CK-666 supports the view that formins promote F-actin dynamics in a pathway parallel to Arp2/3. Moreover, in contrast to Arp2/3, inhibition of formins leaves F-actin turnover rate in PC spines unchanged but dampens spine size fluctuations. Finally, PC spines become more elongated upon interfering with Arp2/3 or WRC (Table 1), consistent with formin-mediated polymerization of linear F-actin remaining. Along this line, depletion of MTSS1, a positive regulator of Arp2/3-mediated F-actin formation and negative regulator of formin DAAM1, also leads to longer PC spines (Saarikangas et al., 2015; Kawabata Galbraith et al., 2018).

We measured an increased size of PC spines upon dominant-negative inhibition of WRC (Figure 6E) and upon acute inhibition of Arp2/3 or formins (Figures 7H,L). This is surprising, given that in hippocampal neurons Arp2/3 is essential for the development of bigger, mature spines (Spence et al., 2016). In part, the distinct outcome may be due to different approaches taken (e.g., acute inhibition vs. genetic depletion). However, we propose that differences in the inherent properties of PC spines compared to hippocampal spines are also a major factor. For example, PC spines form independently of presynaptic input following an intrinsic program (Sotelo, 1978). Moreover, unlike hippocampal spines, PC spines do not undergo drastic morphological changes during plasticity (Sdrulla and Linden, 2007). Finally, in addition to WRC and Arp2/3, also specific F-actin regulators exist in PCs that are not found in other neurons (Miyagi et al., 2002; Sekerkova et al., 2003). Increased PC spine size might affect second messenger signaling within these microcompartements, leading to alteration in synaptic plasticity at PF-PC synapses.

Our data suggest a model where myosin XVI activates the WRC-Arp2/3 pathway in PC spines in order to affect F-actin turnover (Figure 9E). First, *Myo16* depletion via knockout or PC-specific knockdown phenocopies interference with the WRC-Arp2/3 pathway in terms of accelerated F-actin turnover rate in PC spines (Table 1). Second, there was no additive effect of *Myo16* knockout on F-actin turnover upon CK-666 treatment (Figures 7E,F), indicating that the myosin acts through Arp2/3. Third, the model is also supported by the known physical interaction of myosin XVI with the WRC (Yokoyama et al., 2011) and by the localization of both GFP-MYO16 and the WRC component WAVE1 to PC spines (Figures 2, 5). In contrast to interference with WRC and Arp2/3, however, depletion of myosin XVI did not alter the relative size of the mobile F-actin pool and spine circularity (Table 1). This is consistent with the existence of alternative, myosin XVI-independent ways of activating WRC-Arp2/3. Similar to certain synaptic scaffolding proteins (e.g., IRSp53, nArgBP2; Takenawa and Suetsugu, 2007; Lee et al., 2016) and transmembrane proteins (e.g., neuroligins, protocadherins; Chen et al., 2014), myosin XVI might recruit the WRC at specific sites and facilitate its local activation. Nevertheless, our data argue against a general requirement of the myosin for localizing the WRC to spines, as WAVE1 is still present in spines of *Myo16* knockout cerebellum *in situ*. Myosin XVI might also function by bringing PP1c and/or PI3K in close proximity to the WRC in PC spines, thereby helping to regulate the local phosphorylation status – and thus activity – of the WRC.

Genetic ablation of Arp2/3 and WRC components leads to deficits in hippocampal synaptic transmission and to behavior deficits (Soderling et al., 2003, 2007; Kim et al., 2013; Spence et al., 2016). Therefore, also disrupted postsynaptic F-actin turnover caused by *Myo16* knockout may result in altered synaptic targeting of neurotransmitter receptors or altered spine morphology. However, our analyses did not reveal changes in PC spine density or spine morphology upon *Myo16* knockout. Moreover, subcellular fractionation and measurement of mEPSC amplitudes under basal activity indicate that synaptic AMPA receptor numbers are normal in the absence of myosin XVI. Notably, it has been suggested that the late phase of long-term depression in PCs requires actin polymerization (Smith-Hicks et al., 2010). Thus, while we did not detect alterations in postsynaptic morphology or function in *Myo16^–/–^* cerebellum, it is possible that myosin XVI-dependent actin turnover in PC spines specifically affects synaptic plasticity at PF-PC synapses. Importantly, the absence of a deficit in the rotarod test does not rule out that PC-dependent motor learning impairment is present in *Myo16* knockout mice and may be detected using other tests (compare e.g., with Ha et al., 2016).

Both ultrastructural analysis of the cerebellar molecular layer and mEPSC measurements pointed toward a striking presynaptic deficit at *Myo16^–/–^* PF-PC synapses. While synapse density was unchanged, a longer mEPSCs inter-event interval was observed, consistent with less spontaneous fusion events of synaptic vesicles in the absence of myosin XVI. This corroborates the reduction in presynaptic vesicle numbers visualized via electron microscopy. Notably, F-actin is also enriched at presynaptic sites, and presynaptic actin dynamics are thought to play a role for synaptic vesicle fusion (Rust and Maritzen, 2015). Moreover, *Wave1* knockout leads to altered morphology of axon terminals (Hazai et al., 2013), and we observed that WAVE1 localizes presynaptically at molecular layer synapses (Figure 5C). This raises the possibility that myosin XVI regulates actin dynamics at the presynaptic side to promote normal terminal size and synaptic vesicle numbers (Figure 9E). Future experiments will need to test this possibility.

Interference with the regulation of F-actin dynamics may lead to behavioral alterations. For example, *Wave1* knockout mice display motor coordination problems (Soderling et al., 2003). Moreover, an acute requirement of Arp2/3 activity for fear memory formation was demonstrated by microinjecting CK-666 into the lateral amygdala of live rats during fear conditioning (Basu et al., 2016). Since we observed altered F-actin turnover in PC spines and altered synaptic transmission onto PCs upon *Myo16* knockout, we carried out an initial behavioral characterization of *Myo16^–/–^* mice. Notably, while common variations at the *MYO16* locus are associated with ASD (Wang et al., 2009), it is not known if *Myo16* kockout mimics the effect of these alleles. Nevertheless, we included tests for ASD-like behavior. We did not detect phenotypes of *Myo16^–/–^* mice regarding locomotion activity, cerebellum-dependent motor learning, repetitive behavior and social interaction. This suggests that more elaborate future experiments will be needed to determine whether myosin XIV and/or actin turnover play a role for cerebellum-dependent functions such as motor learning or social behavior. Importantly, genetic evidence also indicates links of *MYO16* to schizophrenia (Rodriguez-Murillo et al., 2014) and to bipolar disorder (Kao et al., 2016). Moreover, epigenetic changes at the *MYO16* locus have been detected in depressed patients (Gross et al., 2017) and following learning tasks in mice (Koberstein et al., 2018). Together, this underscores the significance of determining in the future whether myosin XVI is crucial for the function of neuronal circuitry *in vivo*, and whether this involves regulation of F-actin dynamics.

## Materials and Methods

### Plasmids

Prefix “pL7” denotes plasmids carrying the PC-specific *L7* (*Pcp-2*) promoter (Oberdick et al., 1990). Plasmids pL7-mGFP, pL7-mCherry and pL7-mGFP-F-tractin (i.e., pL7-ITPKA-9-52-mGFP) were described previously (Wagner et al., 2011a, b). pL7-ITPKA-9-52-mCherry is identical to pL7-ITPKA-9-52-mGFP except mGFP being replaced by mCherry. To generate pL7-FusionRed for expressing a red fluorescent volume marker in PCs, the FusionRed ORF (Shemiakina et al., 2012) was released from pFusionRed-C vector (#FP411, Evrogen, RU) using NheI, BglII restriction enzymes and ligated with vector backbone obtained by NheI, BglII digest of pL7-mGFP. Plasmid pL7-mGFP-actin was created by inserting a BglII, BamHI fragment encoding human β-actin from pPA-TagRFP-actin (#FP813, Evrogen, RU) in proper orientation into the BglII-site of pL7-mGFP. To generate plasmid pL7-FRED-WAVE1ΔVCA encoding WAVE1 lacking the VCA domain and fused at its N-terminus to FusionRed, a 1503 bp DNA fragment containing mouse *Wasf1* sequence (XM_006512932.2, nucleotides 468 to 1955) flanked by a BglII site at the 5′-end and a stop codon and SalI site at the 3′-end (i.e., 5′-agatct-CCGTTGGTG…AGTGACGCA-tgagtcgac, *Wasf1* sequence in upper case letters) was synthesized (Eurofins Genomics GmbH), digested with BglII and SalI, and ligated with vector backbone obtained by BglII, SalI digest of pL7-FusionRed. Plasmid pL7-mGFP-Myo16 encodes myosin XVI full length heavy chain (XP_006508842.1) fused at its N-terminus to GFP and corresponds to *Mus musculus Myo16* transcript variant X2 (XM_006508779.3) nucleotides 658-6417 cDNA inserted at the BglII, SalI sites of pL7-mGFP. Plasmid pCMV-mGFP-Myo16 was constructed by releasing a 6520 bp fragment comprising the mGFP-Myo16 sequence via NheI, BamHI digest from pL7-mGFP-Myo16. This fragment was ligated into the vector backbone obtained by NheI, BamHI digestion of pFusionRed-C. To construct plasmids for CRISPR/Cas9 sgRNA expression, the web-based tools CRISPRdirect^1^, Optimized CRISPR design^2^ and CCTop^3^ were used to identify guide RNA (gRNA) target sequences flanking *Myo16* exon 3 (5′-TGCTTCAACTCTTGAAGGAGGGGGCAGATCCACA CACTCTCGTGTCCTCAGGAGGGTCTTTGCTACACCTG-3′) on chromosome 8. The selected gRNA target sequences (plus their PAM sequence in lower case letters) were TGCAATTTGCGAAGACCTAC-tgg (gRNA1), ACAATAGCTGTAGGGGCCGT-ggg (gRNA2) and AAGGGCCACGTAGTAACACC-tgg (gRNA3). 5′-phosphorylated oligonucleotides comprising the gRNA target sequence and its reverse complement (see Table 2) were annealed and ligated into the backbone of the pUC57-sgRNA expression vector (Shen et al., 2014) obtained by BsaI digestion, resulting in plasmids pUC57-M16upEx3-sgRNA, pUC57-M16up2Ex3-sgRNA, and pUC57-M16dwEx3-sgRNA. The pUC57-sgRNA expression vector was a gift from Xingxu Huang (Addgene plasmid #51132; RRID: Addgene_51132)^4^. Plasmids for expressing *Myo16* miRNA knockdown constructs under control of *CMV* promoter were termed pcDNA^TM^6.2-GW/EmGFP-miR-*Myo16*-KD1, -KD2, -KD3, -KD4, -KD5 and -scrambled, and were constructed as follows: Target sequences were selected using Invitrogen’s web-based RNAi Designer tool and mouse *Myo16* cDNA sequence BC151049.1. Five predicted perfect candidate target sequences (KD1–KD5) and a scrambled control (KD1 scrambled)^5^ were chosen (see Table 3) and inserted into pcDNA^TM^6.2-GW/EmGFP-miR using the BLOCK-iT^TM^ Pol II miR RNAi Expression Vector Kit (K4935-00, Invitrogen/Thermo Fisher Scientific) according the manufacturer’s instructions. For this purpose, complementary oligonucleotides were annealed (KD1 forward, KD1 reverse, etc., see Table 2). Based on a previously described approach (Alexander and Hammer, 2016), we created plasmids expressing miRNA knockdown constructs under *L7* promoter control. Toward that end, DNA fragments containing the KD/scrambled sequence embedded in the miRNA backbone were PCR amplified from the respective pcDNA^TM^6.2-GW/EmGFP-miR-*Myo16*-KD/scrambled plasmids using oligonucleotides L7sc_F and L7sc_R (Table 2). NheI and NotI-digested PCR fragments were inserted into the NotI/NheI sites of pL7-FusionRed to create pL7-miR-*Myo16* KD3 FRED, pL7-miR-*Myo16* KD5 FRED, and pL7-miR-*Myo16* scrambled FRED. Constructs were verified via restriction digests and sequencing.

**TABLE 2:** Oligonucleotides used in this study.

**Name**	**Sequence (5′ to 3′)**
gRNA1-fw	taggTGCAATTTGCGAAGACCTAC
gRNA1-rv	aaacGTAGGTCTTCGCAAATTGCA
gRNA2-fw	taggACAATAGCTGTAGGGGCCGT
gRNA2-rv	aaacACGGCCCCTACAGCTATTGT
gRNA3-fw	taggAAGGGCCACGTAGTAACACC
gRNA3-rv	aaacGGTGTTACTACGTGGCCCTT
FW1	ATCGTGGGCAAGGGTTAATG
RV1	GCGAGCTGAGACTTGACATTC
FW2	CACGTAACAGGTTTGGGCACAAAG
RV2	TAGCGACACCTGTCACCTGAAATG
KD1 forward	TGCTGTGCTGAAGCCAATTACATTCAGTTTTGGCCACTGACTGACTGAATGTATGGCTTCAGCA
KD1 reverse	CCTGTGCTGAAGCCATACATTCAGTCAGTCAGTGGCCAAAACTGAATGTAATTGGCTTCAGCAC
KD2 forward	TGCTGTAGTGCAGTGAACTGAATGTCGTTTTGGCCACTGACTGACGACATTCATCACTGCACTA
KD2 reverse	CCTGTAGTGCAGTGATGAATGTCGTCAGTCAGTGGCCAAAACGACATTCAGTTCACTGCACTAC
KD3 forward	TGCTGTACACACTCTGTTTGCTCTTGGTTTTGGCCACTGACTGACCAAGAGCACAGAGTGTGTA
KD3 reverse	CCTGTACACACTCTGTGCTCTTGGTCAGTCAGTGGCCAAAACCAAGAGCAAACAGAGTGTGTAC
KD4 forward	TGCTGAGTAATGTCTGCCAGAGATTTGTTTTGGCCACTGACTGACAAATCTCTCAGACATTACT
KD4 reverse	CCTGAGTAATGTCTGAGAGATTTGTCAGTCAGTGGCCAAAACAAATCTCTGGCAGACATTACTC
KD5 forward	TGCTGATAAGAGCCACTGAGCTTCGTGTTTTGGCCACTGACTGACACGAAGCTGTGGCTCTTAT
KD5 reverse	CCTGATAAGAGCCACAGCTTCGTGTCAGTCAGTGGCCAAAACACGAAGCTCAGTGGCTCTTATC
Scrambled FW	TGCTGatactcattatcgacggacatGTTTTGGCCACTGACTGACATGTCCGTATAATGAGTAT
Scrambled RV	CCTGatactcattatacggacatGTCAGTCAGTGGCCAAAACATGTCCGTCGATAATGAGTATC
L7sc_F	ATTAGCGGCCGCTAAGCACTTCGTGGCCGTC
L7sc_R	TAAAGCTAGCCCCGGTAAACAAGGTACACTC

**TABLE 3:** *Myo16* knockdown target sequences.

**Name**	**Sequence (5′ to 3′)**
KD1	TGAATGTAATTGGCTTCAGCA
KD2	GACATTCAGTTCACTGCACTA
KD3	CAAGAGCAAACAGAGTGTGTA
KD4	AAATCTCTGGCAGACATTACT
KD5	ACGAAGCTCAGTGGCTCTTAT
Scrambled	ATGTCCGTCGATAATGAGTAT

### Antibodies

A polyclonal MYO16 antibody (25104-1-AP, Proteintech) was employed. Before usage, the antibody solution was incubated overnight at 4°C with 2% (w/v) brain powder prepared from *Myo16^–/–^* mice to block unspecific interactions. Subsequently, the mixture was centrifuged (30,000 × *g*, 30 min, 4°C) and the supernatant was used at a dilution of 1:600 for Western blot detection. To prepare the knockout brain powder, brains were washed in 0.8% NaCl before adding 1 ml buffer A (1 mM EGTA, 100 mM MES, 0.5 mM MgCl_2_, pH 6.5 with NaOH) per 1 mg brain tissue and homogenization with Potter S Homogenizer (Sartorius AG). After centrifugation (150,000 × g, 1 h, 4°C), the pellet was resuspended in acetone. After 10 min of stirring, the precipitate was let sink down, mixed with fresh acetone and stirred again. After another repeat with fresh acetone, the mixture was filtered to recover the precipitate. After drying overnight, the brain powder was stored at -80°C. In addition, the following antibodies were used in this study at the indicated dilutions (WB, Western blot; IHC, immuno-histochemistry; IEM, immuno-electron microscopy): anti-α-Tubulin (mouse, clone DM1A, T9026, Sigma-Aldrich; WB 1:1,000 – 1:10,000), anti-TUBA4A (rabbit, 177479, Abcam, WB 1:2,000-1:5,000), anti-GFP (rabbit, G1544, Sigma-Aldrich; WB 1:250 – 1:500), anti-WAVE1 (rabbit, WP1731, ECM Biosciences; WB 1:1,000, IHC 1:100, IEM 1:100), anti-Calbindin-D-28K (mouse, C9848, Sigma-Aldrich; IHC 1:200), anti-GluA1 (rabbit, AB1504, Millipore, WB 1:500), anti-GluA2 (mouse, MAB397, Millipore, WB 1:500), anti-PSD-95 (mouse, MA-1-046, Thermo, WB 1:500), anti-GABA_A_ alpha1 (guinea pig, 224205, Synaptic Systems, WB 1:500), anti-NLGN2 (rabbit, 129203, Synaptic Systems, WB 1:500, IHC 1:300), anti-GAPDH (mouse, GTX28245, Genetex, WB 1:1,000-1:2,500), anti-VGLUT1 (guinea pig, AB5905, Millipore, IHC 1:200), anti-Shank2 (rabbit, 162202, Synaptic Systems, IHC 1:300), peroxidase-conjugated anti-rabbit (donkey, 711-036-152, Jackson Immuno Research; WB 1:10,000), peroxidase-conjugated anti-mouse (donkey, 715-036-151, Jackson Immuno Research; WB 1:10,000), Alexa488-conjugated anti-rabbit (711-545-152, Jackson Immuno Research; IHC 1:1,000), Alexa546-conjugated anti-mouse (A11029, Invitrogen/Thermo Fisher Scientific; IHC 1:1,000), and biotinylated anti-rabbit IgG (BA-1000, Vector Laboratories, IEM 1:1,000).

### Mice, Genotyping

Wild type (WT) mice were C57BL/6J (B6). Two novel mouse lines carrying distinct *Myo16* knockout alleles and termed B6-*Myo16^*em*2*Hhtg*^/J* (“S line”; 210 bp deletion) and B6-*Myo16^*em*3*Hhtg*^/J* (“L line”; 269 bp deletion) were generated using CRISPR/Cas9. Both deletions comprise exon 3 of *Myo16* and are thus predicted to lead to a premature stop codon after 77 amino acid residues of MYO16. Plasmids pUC57-M16upEx3-sgRNA, pUC57-M16up2Ex3-sgRNA, and pUC57-M16dwEx3-sgRNA, encoding sgRNA1, sgRNA2, and sgRNA3, respectively, under control of the *T7* promoter, were linearized with DraI and used for *in vitro* transcription using the HiScribe^TM^ T7 High Yield RNA Synthesis Kit (E2040S, New England Biolabs) according to the manufacturer’s instructions. Transcripts were subsequently purified with the MEGAclear^TM^ Transcription Clean-Up Kit (AM1908, ThermoFisher Scientific) according to the manufacturer’s instructions. Pronuclear injection into 1-cell stage embryos obtained from superovulated C57BL6/J mice was performed according to standard protocols using 5 ng/μl for each sgRNA and 16 ng/μl Cas9 protein (M0641T, New England Biolabs). Injected embryos were implanted into F1 foster mothers (C57BL6/J x CBA). PCR genotyping (see below) and sequencing of PCR products identified a founder mouse (#43) that carried both mutant alleles and inherited either one or the other allele, giving rise to both the S and L lines. Gross observation did not reveal phenotypic differences between WT, S and L lines. Moreover, brain size of homozygous *Myo16* knockout mice was indistinguishable from WT littermates (distance Bregma to Lambda, *Myo16^+/+^*: 6.4 ± 0.7 mm, *Myo16^–/–^*: 7.8 ± 1.3 mm; brain area, *Myo16^+/+^*: 88.5 ± 14.6 mm^2^, *Myo16^–/–^*: 114.1 ± 34.6 mm^2^; cerebellar area: *Myo16^+/+^*: 23.3 ± 3.3 mm^2^, *Myo16^–/–^*: 26.7 ± 7.3 mm^2^; mean ± SD, *n* = 3; *p* = n.s., Student’s *t*-test). PCR genotyping was performed to distinguish among absence, heterozygous presence or homozygous presence of the *Myo16* knockout alleles. Genomic DNA was obtained by digesting tail biopsies or, for neuronal cultures, embryo tissue with QuickExtract^TM^ DNA Extraction Solution (Epicentre) for 30 min at 65°C, followed by incubation for 10 min at 97°C. PCR was performed using primers FW2 and RV2 (see Table 2), Taq DNA Polymerase (Roche # 04728874001), and reaction conditions of 94°C for 2 min; 35 times repeat of (94°C for 10 s, 67°C for 20 s, 72°C for 10 s); followed by 72°C for 3 min. DNA fragments were separated on 1.2–2.0% agarose gels and imaged using a UV transillumination/digital camera system (INTAS Science Imaging Instruments). *Myo16* knockout alleles yield a 191 bp (S line) or 132 bp (L line) fragment, the WT allele yields a 401 bp fragment (Figure 1B). Alternatively, PCR was performed as above, but using primers FW1 and RV1 (see Table 2), and reaction conditions of 94°C for 2 min; 35 times repeat of (94°C for 10 s, 68°C for 20 s, 72°C for 25 s); followed by 72°C for 5 min. Here, *Myo16* knockout alleles yield a 519 bp (S line) or 460 bp (L line) fragment, the WT allele yields a 729 bp fragment. Homozygous *Myo16* knockout mice (*Myo16^–/–^* mice) were obtained by mating heterozygous mice. Absence of MYO16 protein in *Myo16^–/–^* mice was confirmed by Western blot using pre-adsorbed anti-MYO16 antibody and extracts of cerebellum (Figure 1C) and hippocampus of young and adult mice.

### Protein Extracts, Fractionation, and Western Blot Analyses

For detection of MYO16 and WAVE1 in cerebellar crude extracts (Figures 1C, 5A), single cerebella were lysed in ice cold lysis buffer (150 mM NaCl, 1% IGEPAL^®^ CA-630 [I8896, Sigma-Aldrich], 50 mM Tris–Cl pH8.0, protease inhibitors [cOmplete^TM^ Protease Inhibitor Cocktail; #04693116001, Roche]) using 12 strokes with Potter S Homogenizer (Sartorius AG). The homogenate was centrifuged at 600 × g for 10 min, 4°C and the supernatant was mixed with 20% (v/v) loading dye (5% β-mercaptoethanol, 0.02% bromophenol blue, 30% glycerol, 10% sodium dodecyl sulfate [SDS], 250 mM Tris–Cl pH 6.8) and denatured (10 min, 97°C).

Subcellular fractionation of cerebellar extracts (Figures 1H,I) to yield a crude extract (S1), a membrane-enriched fraction (P2), a cytosolic fraction (S2), and a synaptosomal fraction (SYP) was carried out by lysing a complete cerebellum in Sucrose 1 buffer (320 mM sucrose, 1 mM NaHCO_3_, 1 mM MgCl_2_, 500 μM CaCl_2_, 1 μM PMSF) in the presence of EDTA-free protease inhibitors (cOmplete Tablets, EASYpack, 04693132001, Roche) and phosphatase inhibitors (PhosSTOP, EASYpack, 04906837001, Roche), by potterization in 2 ml teflon tubes at 800 rpm and 12 strokes. S1 post-nuclear fraction was obtained by centrifugation of the total lysate at 1,400 × *g* for 10 min at 4°C. A fraction of S1 was conserved for further analysis and the rest was centrifugated at 13,800 × g for 10 min at 4°C to obtain S2 soluble and P2 membranous fractions. S2 fraction was conserved and P2 was resuspended in Sucrose 2 buffer (320 mM sucrose, 1 mM NaHCO_3_). P2 was shaken for 20-30 min at 4°C. Sucrose gradient for synaptosomal purification was achieved by sequentially adding three distinct layers of 1.2 M, 1 M, and 0.8 M sucrose in the presence of NaHCO_3_. A part of the P2 fraction was conserved for analysis and 400 μl were placed on top of the gradient and centrifugated at 22,000 rpm using SW40 Ti rotor for 2 h at 4°C. Synaptosomes were isolated from the interface between 1.2 and 1 M fractions of the gradient using a 1 ml syringe. In order to assess myosin XVI localization in synaptosomes (Figure 2A), an identical protocol was used, however, omitting phosphatase inhibitors and PMSF from sucrose buffer 1 and, occasionally, NaHCO_3_ from sucrose gradient. Protein concentration was quantified with Pierce^TM^ BCA Protein Assay kit (#23227, Thermo Fisher Scientific), and identical amounts of proteins were mixed with loading dye and denatured as above.

Cerebellar crude extracts and fractions were separated via standard SDS-PAGE using 6–15% gels. Spectra^TM^ Multicolor High Range Protein Ladder (26625, Thermo Fischer Scientific) and BlueStar Plus prestained protein markers (MWP04 and MWP03, Nippon Genetics) were used as size standards. Proteins were transferred onto methanol-activated polyvinylidene difluoride membranes (PVDF; Immobilon-P, #IPVH00010, Merck Millipore) using transfer buffer 1 (39 mM glycine, 48 mM Tris–Cl pH 8.3, 0.037% SDS, 20% [v/v] methanol) and semi-dry blotter V20-SDB (SCIE-PLAS, Cambridge, United Kingdom) for 2 h or transfer buffer 2 (25 mM Tris–Cl pH 8.3, 192 mM glycine and 20% [v/v] methanol) and wet blotting via Mini-Protean Tetra Cell system (Biorad). Subsequently, the membrane was blocked with TBST (20 mM Tris–Cl pH 7.6, 150 mM NaCl, 0.1% Tween20) containing 5% (w/v) non-fat dry milk or 3% bovine serum albumin (BSA) for 1 h at room temperature (RT). Incubations with primary and horse-radish peroxidase-coupled secondary antibodies were also carried out in blocking solution.

For *Myo16* knockdown in HEK cells, HEK293 cells (CRL-1573, ATCC) grown in DMEM, high glucose, GlutaMAX^TM^ Supplement (61965026, ThermoFisher Scientific) containing 20% serum to a density of ∼75% were co-transfected using the calcium phosphate method (Gromova et al., 2018) with plasmid pCMV-mGFP-Myo16 and a knockdown plasmid (pcDNA^TM^6.2-GW/EmGFP-miR-*Myo16*-KD1, -KD2, -KD3, -KD4, -KD5, or -scrambled), as indicated. Cells were harvested 48 h after transfection and lysed on ice by pipetting up and down in PBS (137 mM NaCl, 2.7 mM KCl, 10 mM Na_2_HPO_4_, 1.8 mM KH_2_PO_4_, pH 7.4) containing 1% Triton X-100, protease inhibitors (cOmplete^TM^, EDTA-free Protease Inhibitor Cocktail; #04693132001, Roche) and phosphatase inhibitors (PhosSTOP^TM^; #04906845001, Roche). After centrifugation at 1,000 × g for 5 min at 4°C, the supernatant was removed and protein concentration was quantified with Pierce^TM^ BCA Protein Assay kit (#23227, Thermo Fisher Scientific). Identical amounts of protein were separated via SDS-PAGE using 8–15% gels. Western blots were performed as above except that loading dye contained 10% β-mercaptoethanol, size standard was Precision Plus Protein^TM^ Dual Color Standard (1610374, Biorad), and wet blotting was used with transfer buffer 2.

Chemiluminiscence detection was performed using Immobilon Western HRP Substrate (#WBKLS0500, Merck Millipore) and CCD camera-based ChemoStar ECL detection system (ChemoCam Imager ECL Typ HR 16-3200, INTAS Science Imaging Instruments, Göttingen, GER). Signals were quantified using Fiji image processing software (Schindelin et al., 2012).

### Dissociated Cerebellar Cultures Containing Purkinje Cells

Preparation and transfection of cerebellar cultures containing PCs was done as described (Wagner et al., 2011b), with minor modifications. Briefly, C57BL/6J X C57BL/6J or *Myo16*^±^ X *Myo16*^±^ matings were used to obtain E17-18 mouse embryos from females after euthanization with CO_2_ and cervical dislocation. Embryonic brains were isolated in ice-cold modified Hank’s balanced salt solution (MHS; HBSS without calcium and magnesium, Gibco^TM^ 14170088, Thermo Fisher Scientific) and treated individually during the whole procedure. In case of *Myo16* knockout cultures, *Myo16^–/–^*, *Myo16*^±^ and *Myo16^+/+^* brains were distinguished using PCR genotyping using embryo head tissue while the isolated brains were stored in Hibernate^TM^-E medium (Gibco^TM^ A1247601, Thermo Fisher Scientific) up to 6 h. The cerebellar primordium was minced and digested for 20 min at 30°C using 7 U papain (P3125, Sigma-Aldrich). After addition of heat-inactivated fetal bovine serum (FBS; Gibco^TM^ 10082139, Thermo Fisher Scientific), cells were triturated in MHS containing 12 mM MgSO_4_ (M2643, Sigma-Aldrich) and 5 U/ml DNAse I (Roche 04716728001, Sigma-Aldrich) and filtered through a nylon mesh (180 μm pore size, #NY8H04700, Millipore). After a wash in MHS, all cells from one cerebellum were nucleofected in a single reaction using Mouse Neuron Nucleofector^®^ Kit (VPG-1001; Lonza; nucleofection program O-003) according to manufacturer’s instructions. The transfected cells were then mixed with untransfected cells from one WT cerebellum resuspended in 300 μl DFM (see below) supplemented with 10% (v/v) FBS. DFM consisted of Dulbecco’s Modified Eagle’s Medium/Nutrient Mixture F-12 Ham (D6434, Sigma-Aldrich) supplemented with 1x GlutaMAX (Gibco^TM^ 35050-038; Thermo Fisher Scientific), 100 μM putrescine dihydrochloride (P5780, Sigma-Aldrich), 30 nM Na_2_SeO_3_ (S5261, Sigma-Aldrich), 40 nM progesterone (P7556, Sigma-Aldrich), 0.77 nM L-3,3′,5-tri-iodothyronine (T2877, Sigma-Aldrich), 2 μM cytosine β-D-arabinofuranoside (C6645, Sigma-Aldrich), 200 μg/ml apo-transferrin (T1147, Sigma-Aldrich), 100 μg/ml BSA (A3156, Sigma-Aldrich), and 20 μg/ml insulin (I0516; Sigma-Aldrich). The whole cell mixture was plated onto the glass surface (14 mm diameter) of a single glass bottom dish (D35-14-1.5-N, Invitro Scientific) coated with poly-L-ornithine hydrobromide (P4638, Sigma-Aldrich), thus resulting in a single culture of transfected cells per cerebellum. Cultures were kept in an incubator (37°C, 5% CO_2_, saturated humidity). At 1.5 h after plating, 1.8 ml DFM containing 5 μg/ml gentamicin (Gibco^TM^ 15710049; Thermo Fisher Scientific) were added per dish, and 4–36 h after plating 1.5 ml of culture medium were replaced with fresh DFM/gentamicin. Subsequently 1 ml of medium was replaced by fresh DFM/gentamicin once per week.

### Live Cell Microscopy

Cultured PCs were observed using a spinning disk confocal microscope (Visitron Systems GmbH) consisting of an inverted microscope (Nikon Ti-E) equipped with a spinning disk (Yokogawa), solid state lasers (405, 488, 561, and 647 nm), a 100× objective, EM-CCD cameras (Hamamatsu Photonics), autofocus system (Nikon TI-ND6-PFS), a multi-point FRAP/photo-activation module (VisiFRAP, Visitron Systems GmbH), and an incubation chamber for controlled environmental conditions (37°C, 5% CO_2_). During imaging, cultures were kept in their conditioned growth medium. Images shown in Figures 2B,D were obtained by recording *Z*-stacks (0.3 μm *Z*-plane distance) and subjecting them to noise removal (de-noise, low pass filter; VisiView^®^ software, Visitron Systems GmbH). Maximum projections were generated and stitched using Fiji/MosaicJ (Thevenaz and Unser, 2007; Schindelin et al., 2012). Images shown in Figures 2C,E,F, as well as Supplementary Movies S1–S3 were obtained recording at a frame rate of 0.5/s, followed by noise removal (de-noise, low pass filter; VisiView^®^ software, Visitron Systems GmbH). Subsequently images were processed in Fiji (Schindelin et al., 2012) using bleach correction (histogram matching) and generation of a three frame rolling average image.

### Fluorescence Recovery After Photobleaching

To measure F-actin turnover in PC spines, FRAP experiments were performed with cultured PCs expressing GFP-actin at 14–15 DIV using the spinning disk microscope with a 100× objective. Images were obtained using 488 nm excitation and an exposure time of 500 ms. In each trial, following five frames of starting point recording (frame rate of 1/s), the GFP-actin fluorescence signal of six single spines on a PC was bleached by directing 405 nm laser light to a circular region (12 pixel diameter) placed on the spine (∼2 s total bleach time for six spines). Imaging was resumed immediately after and continued for 145 s at a frame rate of 1/s. If indicated, the following compounds were added to the culture medium prior to carrying out FRAP recordings: jasplakinolide (#420127-50UG, Merck; added to final concentration of 1 μM; FRAP performed 10–60 min after addition), CK-666 (#3950, Tocris Bioscience; added to final concentration of 200 μM; FRAP performed within 90 min after addition), SMIFH2 (S4826, Sigma-Aldrich; added to final concentration of 40 μM; FRAP performed 5–50 min after addition, as with combination of SMIFH2 and CK-666).

GFP-actin fluorescence intensity signal in bleached spines was quantified using Fiji (Schindelin et al., 2012; Schneider et al., 2012). Intensity was measured from a region of constant size encompassing the spine throughout the duration of the experiment. To determine recovery of the bleached fluorescence signal, the intensity measured in each frame was reduced by the intensity remaining in the first frame after bleaching, yielding baseline-corrected intensity values *F*_b_. To correct for overall bleaching during the experiment, *F*_b_ was multiplied with a bleach factor (*y*) calculated for each trial and time point, yielding bleach-corrected intensity values (*F*_bc_). To obtain *y*, fluorescence intensity in each frame was measured from part of the cell that included only spines not targeted by FRAP bleaching. After background correction of the measured intensities, bleach factor y was determined for each time point using Excel (Microsoft) by fitting a curve described by the exponential equation

$$y={\text{at}}^{b}$$

(*a* and *b*, fitted variables; *t*, time) to the values obtained by dividing the initial, background-corrected intensity by the background-corrected intensity at each time point. The mean of *F*_bc_ (the bleach-corrected intensity values) before FRAP bleaching was normalized to 100% (*F*_0_), and *F*_bc_ values at each time point after bleaching were expressed relative to *F*_0_ (GFP-actin fluorescence,% of initial), yielding the fluorescence recovery curve. For each independent experiment (i.e., for each culture of transfected PCs derived from an individual embryo), a mean recovery curve was calculated from the recovery curves of roughly 50 spines on average, with up to six curves originating from a single FRAP trial. To calculate plateau and time constant τ of GFP-actin fluorescence intensity recovery in spines, the mean recovery curves from each experiment were fitted to the equation

y=P⁢(1-e⁢x⁢p⁢(-k⁢x))

*y*, GFP-actin fluorescence intensity; *x*, time; *P*, plateau; *k*, inverse of τ. In case a mean recovery curve showed an intensity decrease of more than five percentage points at any time during the recovery phase, the values following the pre-decrease maximum value were ignored when fitting the equation. If this led to less than 70 s of recovery time that could be fitted, the data were excluded entirely from analysis. Cells that were immotile and stiff (i.e., apparently dead) during FRAP recording were also excluded from analyses. Spines were excluded if the fluorescence signal was oversaturated, if the spine overlapped with another spine during the 150 s of recording, or if the fluorescence signal was not bleached close to background level.

### Spine Morphology and Dynamics of Live PCs

The following parameters were determined using Fiji and the time-lapse movies of GFP-actin recorded during the FRAP experiments. 50 spines (five spines from ten PCs) that were not FRAP-bleached were analyzed for each condition. The area covered by randomly selected, individual spines was determined in each frame by selecting a threshold at the level of dendritic shaft GFP-actin signal intensity, followed by an automatic creation of a smoothened region of interest (ROI) around the above-threshold spine GFP-actin signal in each frame. The “spine area” was determined by averaging the ROI area of the first three frames (1 s recording interval) for each spine. The variation of the spine area during 2.5 min (150 frames of recording), denoted as “SD (area over time),” is the standard deviation of the relative area (% of initial) over 150 frames, with the average area of the first three frames representing 100%. A circularity index (CI) was calculated from the ROI using the formula

$$\mathrm{CI}=\frac{4\,\pi \,a}{p^{2}}$$

(*a*, area; *p*, perimeter) (Rubio et al., 2011) and is given as the average value of the first three frames (1 s recording interval) for each spine. The variation of the GFP-actin signal intensity in spines during 2.5 min (150 frames of recording), denoted as “SD (intensity over time),” is the standard deviation of the relative fluorescence intensity (% of initial) over 150 frames, with the average intensity of the first 20 frames representing 100%. Spine GFP-actin signal intensity was determined for each frame by measuring the integrated intensity within the above determined ROI, followed by background subtraction, bleaching correction and calculation of a 20 frame rolling average intensity value.

### Immuno-Histochemistry and Immuno-Electron Microscopy

For WAVE1 immuno-histochemistry (Figure 5B), ∼6 month old adult mice were anesthetized by a mixture of ketanest and rompun and transcardially perfused with 4% paraformaldehyde (PFA) in phosphate-buffer (PB). Brains were post-fixed in PFA/PB (overnight, 4°C) before 50 μm vibratome sections were cut. Brain sections were washed in PBS, incubated for 10 min in 0.5% (w/v) NaBH_4_ in PBS, washed again in PBS, and blocked for 30 min in PBS containing 10% (v/v) horse serum (HS), 0.3% (w/v) BSA and 0.3% (v/v) Triton X-100. Sections were then incubated with primary antibodies diluted in carrier (PBS containing 1% HS, 0.2% BSA and 0.3% Triton X-100) for 24 h at 4°C with gentle agitation, washed in PBS, and incubated for 2 h with secondary antibodies diluted in carrier. After wash in PBS, sections were mounted on glass slides using ProLong^TM^ Gold Antifade Mountant (P36930; Thermo Fisher Scientific), imaged on an Olympus FV1000 confocal laser scanning microscope (60× objective; 0.8 μm *Z*-plane thickness).

Similarily, for immuno-histochemistry shown in Figures 1D–G, 19–26 week old mice were perfused as above. Postfixation was carried out for 48 h, followed by cryoprotection of the brains in 30% Sucrose/PBS. Brains were frozen in TissueTek at -80°C and 40 mm thick sagittal sections were obtained with a cryostat (Cryostar NX70, Thermo Scientific). For staining, sections were washed in PBS and permeabilized 20 min in presence of PBS/0.5% Triton X-100 at RT. Subsequently, sections were washed three times in PBS and blocked with blocking buffer (PBS/10% goat serum/1% BSA) for 1–2 h at RT. Primary antibodies were diluted in Ab incubation buffer (PBS/3% goat serum, 1% BSA/0.05% Triton X-100) and incubated over night in a wet chamber at 4°C before washing the sections three times in PBS, followed by incubation with the secondary antibodies in Ab incubation buffer (1–2 h at RT). After three washes, sections were mounted using Aqua Poly/Mount (18606-20, Polysciences, Inc.) and observed using confocal laser scanning microscopy as above.

Images were processed and analyzed using Fiji (Schindelin et al., 2012). Shank2 clusters were manually counted in a ∼432 um^2^ square area within three sections of the cerebellar molecular layer (identified via calbindin staining) for each animal. NLGN2 clusters were counted in a ∼6000 um^2^ square area spanning the whole molecular layer in five sections per animal using the “Analyze Particles” function.

For Nissl staining, sections were delipidized in ethanol with increasing concentration (70, 95, and 100%) followed by submersion in xylen (30 s per step). Rehydration was performed following the opposite order ending in H_2_O for 4 min. After 3–4 min incubation in cresyl violet stain (0.1% cresyl violet acetate, 2.5% glacial acetic acid) sections were rinsed with dH_2_O for 1 min and 6–7 times in 70% ethanol. Subsequently, a differentiation step was carried out by alternating between 95% ethanol and 95% ethanol with 10% acetic acid. Sections were then dehydrated with consecutive 30 s washes in 95% ethanol, 100% ethanol and xylen before covered with entellan. Images were taken with a stereomicroscope (Stemi 2000C, Zeiss, GER).

For ultrastructural electron microscopy (Figure 8), 40 week old anesthetized mice that previously underwent behavioral testing (see below) were transcardially perfused with 4% PFA (postfixed in a mixture of 4%PFA and 1% glutaraldehyde) in 0.1 M PB at pH 7.4. Similarly, for immuno-electron microscopy (Figure 5C), 38 week old mice were transcardially perfused with a mixture of 4% PFA and 0.1% glutaraldehyde in 0.1 M PB at pH 7.4. Brains were removed and 100 μm thick sagittal sections were cut with a Vibratom (Leica VT 1000S). Thereafter, pre-embedding immuno-electron microscopy was performed as follows: Sections were cryoprotected in 2.3M sucrose and subjected to two cycles of freeze-thaw in liquid nitrogen to aid penetration of immunoreagents into tissue. After rinsing in PBS, sections were incubated with PBS containing 10% HS and 0.2% BSA for 15 min, before being incubated with primary antibody in PBS containing 1% HS and 0.2% BSA overnight. Cells were washed with PBS, then incubated with biotinylated secondary antibody in PBS containing 1% HS and 0.2% BSA for 90 min. After rinsing, they were incubated with ABC (Vector Labs) diluted 1:100 in PBS for 90 min. Sections were washed in PBS and reacted in diaminobenzidine (DAB)-H_2_O_2_ solution (Sigma, St. Louis, United States) for 10 min. Thereafter sections were rinsed three times in 0.1 M sodium cacodylate buffer (pH 7.2–7.4) (Sigma-Aldrich, Buchs, Switzerland) and incubated with 1% osmium tetroxide (Science Services, Munich, Germany) in cacodylate buffer for 20 min on ice. The osmication of sections was followed by dehydration through ascending ethyl alcohol concentration steps and rinsed twice in propylene oxide (Sigma-Aldrich, Buchs, Switzerland). Infiltration of the embedding medium was performed by immersing the tissue first in a mixture of 2:1 of propylene oxide and Epon (Carl Roth, Karlsruhe, Germany), then in a 1:1 mixture, and finally in neat Epon and hardened at 60°C for 48 h. Ultrathin sections (60 nm) were collected and analyzed with an EM902 transmission electron microscope (Zeiss, Germany) equipped with a CCD in lens 2K digital camera and running the ImageSP software (Tröndle, Moorenweis, Germany).

Quantification of synaptic parameters from electron microscopy images was performed using Fiji. For counting molecular layer synapse numbers, 7.086 μm × 7.086 μm images were obtained with 12,000× magnification. 25 consecutive images of the middle third area of the molecular layer were analyzed for each animal, with a total of three animals per genotype. Synapses were counted if they showed a uninterupted outline of the pre- and postsynaptic specialization, ER structure and PSD in spine head and an opposing presynaptic terminal with vesicles. For analyzing spine and presynaptic structural properties, images of the cerebellar molecular layer were taken using 20,000× magnification. Synapses were identified using the same criteria as before.

### Cerebellar Slice Electrophysiology and Biocytin Staining of PCs

Mice used for electrophysiological measurements were 3–4 weeks old. After decapitation of mice under isoflurane anesthesia, the brain was removed into ice-cold slicing medium containing (in mM) 240 sucrose, 2.5 Na_2_HPO_4_, 2 MgSO_4_, 26 NaHCO_3_, 10 D-glucose, and 1 CaCl_2_ which was carbonated continuously (95% O_2_, 5% CO_2_). 200 μm thick sagittal slices of the cerebellum were cut using a vibratome (Leica CT1200S) and left for incubation in artificial cerebrospinal fluid (ACSF) containing (in mM) 124 NaCl_2_, 5 KCl, 1.25 NaHPO_4_, 2 MgSO_4_, 26 NaHCO_3_, 20 D-glucose and 2 CaCl_2_ for 1 h at 37°C and constant carbonation. mEPSCs were measured at 34°C and in the presence of 100 μM picrotoxin (P1675, Sigma-Aldrich), 50 μM (2R)-amino-5-phosphonovalecric acid (D-APV; 79055-68-8, Tocris) and 1 μM tetrodotoxin (TTX; 1078, Tocris). Whole-cell patch clamp recordings were performed with an EPC10 amplifier (HEKA Electronics, Lamprecht, Germany). PCs were visualized using a research Zeiss Axioskop 2 FS plus microscope equipped with a 40x objective. Recording electrodes of 4–5 MΩ, 1.5 mm outer diameter and 0.87 mm inner diameter (1810016, Hilgenberg) were filled with intracellular solution containing (in mM) 120 K-gluconate, 9 KCl, 10 KOH, 3.48 MgCl_2_, 4 NaCl, 10 HEPES, 4 Na_2_ATP, 0.4 Na_3_GTP, 17.5 sucrose and 1 mg/ml biocytin (3349, Tocris) (pH 7.25–7.35 with an osmolality of 295 ± 5). Recordings were excluded from analysis if the noise level exceeded 10 pA. Slices with successfully measured cells were fixed after recording in Histofix (P087.6, Roth), washed with PBS and permeabilized in PBS/0.2% Triton. 20% [w/v] BSA was added for blocking. For staining, Alexa Fluor^®^ 488 streptavidin conjugate (S32354, Invitrogen/Thermo Fisher Scientific) was diluted in PBS/20% BSA (1:1,000). Slices were mounted in Aqua Poly/Mount (18606-20, Polysciences, Inc.) and imaged using a confocal laser scanning microscope (Olympus FV1000) equipped with a 60× objective (488 nm laser line for excitation, 0.2 μm *Z*-plane thickness). Deconvolution was performed using AutoQant (100 deconvolution cycles; Media Cybernetics Inc.). To determine spine density, Imaris filament tracer and Imaris 8.4.1 spine module (Imaris Bitplane) were used.

### Mouse Behavior Analyses

All behavior experiments were performed with a cohort of males of the B6-*Myo16^*em*3*Hhtg*^/J* line backcrossed to C57BL/6J for at least five generations. Heterozygous mice were bred in house to obtain naïve, age-matched *Myo16^+/+^* (11 individuals) and *Myo16^–/–^* (11 individuals) mice for the experiments. Weight did not differ significantly between groups. Animals were housed in groups of littermates (3–5 individuals per cage) in an acclimatized animal vivarium (21 ± 1°C, relative humidity 55 ± 5%) under a 12 h:12 h reversed light/dark cycle and were tested during dark hours. The mice had *ad libitum* access to food and water. At the beginning of the experiments, animals were between 9 and12 weeks old. All experiments were performed blind to genotype.

#### Open Field

The open field apparatus consisted of four identical square (50×50×50 cm) arenas made of white polyvinyl foam material. Four lamps were installed that provided even lightning (50 lux) in each arena and a video camera that was mounted directly above the apparatus. The videos were transmitted to a computer running Ethovision tracking software (Version XT8.5, Noldus Technology, Netherlands). Up to 4 mice were tested at the same time, counterbalanced across genotypes but blind to the experimenter. The mice were introduced to one corner of the arena and were allowed to explore undisturbed for 30 min. Total distance moved, velocity and mean distance to the wall were analyzed in 5 min consecutive time bins.

#### Rotarod

The Ugo Basile Model 47600 (Comerio, VA, Italy) accelerating rotarod for mice was used. The testing area was illuminated diffusely with 30 lux white light. A digital camera was located close to the apparatus to capture videos. The test was performed in two steps. For habituation, up to five subjects were tested simultaneously and placed on the rotating drum at a baseline speed of four rounds per minute (rpm) for up to 180 s. Two trials were performed with an inter-trial interval of 50–60 min. On the same day, three trials were performed for each subject with the speed increasing linearly for 4 min from 4 to 40 rpm. Animals were allowed to stay on the rotating drum for up to 600 s with an inter trial interval of 50–60 min. On the next 5 days each subject was tested in three trials per day of accelerating speed as described above, however, the inter-trial interval was shortened to 10–15 min. The latency to fall was measured. Two capture a second time window, the same animals performed the task again 5 weeks later for seven consecutive days.

#### Marble Burying Test

Two 26×42 cm cages, filled 6 cm high with fine fresh bedding material were used. Cages were closed with a plastic plate leaving space for air circulation. On the bedding, 20 marbles were placed in a regular pattern, covering the whole area. The apparatus was illuminated by diffuse light of 30 lux. A digital camera was located close to the apparatus to capture videos. Each mouse was introduced into the cage and allowed to explore freely for 30 min. Between subjects, the bedding was stirred thoroughly, pressed down to have a plane surface and marbles were again placed on top. The marbles buried were counted manually in 5 min time bins.

#### Social Interaction Test

The social interaction test was performed in a white, compartmentalized box made out of polyvinyl foam material. The box had the dimensions of 61 × 37 cm and was divided twice, resulting in three linked compartments: left (22 × 37 cm), center (17 × 37 cm), and right (22 × 37 cm), with doors in between. In the outer compartments, a round chamber (12 cm diameter, 13 cm high) with a heavy lid was placed. The apparatus was illuminated with 30 lux and a digital camera was mounted directly above the setup. Videos were transmitted to a computer running Ethovision tracking software (Version XT8.5, Noldus Technology, Netherlands) equipped with three-point (nose, body center, and tail) detection settings. After 5 min of habituation in the center compartment, doors were opened and mice were allowed to move freely between all three compartments. Active exploration was scored when the nose of the test mouse was detected within a distance of 2 cm to the round chambers. A WT mouse unknown to the test mouse (novel mouse) was present in one chamber, whereas the second chamber was empty. After 10 min a second, unfamiliar WT mouse (novel mouse) was placed in the empty chamber and the test mouse was allowed to explore for another 10 min. In the first part of the test, time spent with the novel mouse compared to the empty chamber was analyzed. In the second part, the time spent with unfamiliar mouse compared to familiar mouse was analyzed. Discrimination index was calculated as time spent with “novel mouse” divided by the time spent with “empty chamber” and “novel mouse” multiplied by 100 (part 1), or time spent with “novel mouse” divided by the time spent with “familiar mouse” and “novel mouse” (part 2) multiplied by 100.

### Statistics

Analyses were performed using Prism 7.04 (GraphPad Software, Inc.). All data sets were subjected to Shapiro-Wilk normality test. When comparing two groups fulfilling the normality test, *p* values were obtained using Student’s *t*-test (if variance in data sets was the same according to *F* test) or using *t*-test with Welch’s correction (if variance in data sets was different according to *F* test). When comparing two groups not fulfilling the normality test, exact *p* values were obtained using Mann–Whitney test. For comparison of more than two groups that all fulfill the normality test, the presence of significant differences was first evaluated using ordinary one-way ANOVA, followed by Dunnett’s, Tukey’s or Sidak’s multiple comparisons test against the control values. For comparison of more than two groups that not all fulfill the normality test, the presence of significant differences was first evaluated using ordinary Kruskal-Wallis test, followed by Dunn’s multiple comparisons test against the control values. Significance values are indicated in the figures as ^*^p < 0.05; ^∗∗^*p* < 0.01; ^∗∗∗^*p* < 0.001; ^****^p < 0.0001; n.s., not significant. For behavior analyses, data were analyzed using IBM SPSS Statistics (SPSS Inc., Chicago, IL, United States, Version 22). The statistical tests are indicated in the figure legends of each experiment. For statistical significance a type I error rate of *p* = 0.05 was defined, all tests were performed two tailed. For analyzing data measured in different time bins, repeated measures ANOVA with genotype as between subject factor and time (bins) as within subject factor was carried out, followed by a Bonferroni *post hoc* test whenever appropriate.

## Data Availability

The datasets generated for this study are available on request to the corresponding author.

## Ethics Statement

This study was carried out in accordance with the recommendations of the European Community Council Directive (2010/63/EU) and the procedures used were approved by the City of Hamburg (Behörde für Gesundheit und Verbraucherschutz, Lebensmittelsicherheit und Veterinärwesen).

## Author Contributions

WW conceptualized the work and wrote the original draft. All authors contributed to the methodology and wrote, reviewed, and edited the manuscript. MR, FL, SF, MS, and WW contributed to the investigation and formal analysis. IH-B and MK contributed to the resources. MK and WW acquired the funding. SF, JS, and WW supervised the work.

## Conflict of Interest Statement

The authors declare that the research was conducted in the absence of any commercial or financial relationships that could be construed as a potential conflict of interest.

## Footnotes

1 https://crispr.dbcls.jp/

2 http://crispr.mit.edu/

3 https://crispr.cos.uni-heidelberg.de/

4 http://n2t.net/addgene:51132

5 https://www.genscript.com/tools/create-scrambled-sequence

## Supplementary Material

**MOVIE S1 |** Movie depicting GFP-MYO16 (green) in dendritic spines of live Purkinje cells (volume marker; magenta). Time indicated in min:sec; scale bar, 2 μm. Related to Figure 2C.

**MOVIE S2 |** Movie depicting F-actin (F-tractin probe; green) in dendritic spines of live Purkinje cells (volume marker; magenta). Time indicated in min:sec; scale bar, 2 μm. Related to Figure 2E.

**MOVIE S3 |** Movie depicting GFP-MYO16 (green) and F-actin (F-tractin probe; magenta) in dendritic spines of live Purkinje cells. Time indicated in min:sec; scale bar, 2 μm. Related to Figure 2F.

**MOVIE S4 |** Movie depicting an example for FRAP analysis of GFP-actin in Purkinje cell spines. Green circles indicate bleached spines. Time indicated in min:sec; scale bar, 2 μm. Related to Figure 3A.

**MOVIE S5 |** Movie depicting GFP-actin in dendritic spines of live Purkinje cells transfected with control plasmid (pL7-FRED). Time indicated in min:sec; scale bar, 2 μm. Related to Figure 6H.

**MOVIE S6 |** Movie depicting GFP-actin in dendritic spines of live Purkinje cells transfected with a plasmid encoding a dominant-negative version of WAVE1 (pL7-FRED-WAVE1ΔVCA). Time indicated in min:sec; scale bar, 2 μm. Related to Figure 6H.

## Abbreviations

ASD: autism spectrum disorder
DIV: days *in vitro*
F-actin: filamentous actin
FRAP: fluorescence recovery after photobleaching
mEPSCs: miniature excitatory postsynaptic currents
miR: microRNA
PCs: Purkinje cells
PFs: parallel fibers
RNAi: inhibitory RNA
WRC: WAVE regulatory complex.

## References

[B1] Alexander C. J. Hammer J. A. III (2016). Optimization of cerebellar purkinje neuron cultures and development of a plasmid-based method for purkinje neuron-specific, miRNA-mediated protein knockdown. *Methods Cell Biol.* 131 177–197. 10.1016/bs.mcb.2015.06.004 26794514 PMC6699752

[B2] Basu S. Kustanovich I. Lamprecht R. (2016). Arp2/3 and VASP are essential for fear memory formation in lateral amygdala. *eNeuro* 3:ENEURO.0302-16.2016. 10.1523/eneuro.0302-16.2016 27957528 PMC5126706

[B3] Basu S. Lamprecht R. (2018). The role of actin cytoskeleton in dendritic spines in the maintenance of long-term memory. *Front. Mol. Neurosci.* 11:143. 10.3389/fnmol.2018.00143 29765302 PMC5938600

[B4] Bertling E. Englund J. Minkeviciene R. Koskinen M. Segerstrale M. Castren E. (2016). Actin tyrosine-53-phosphorylation in neuronal maturation and synaptic plasticity. *J. Neurosci.* 36 5299–5313. 10.1523/JNEUROSCI.2649-15.2016 27170127 PMC6601809

[B5] Bisi S. Disanza A. Malinverno C. Frittoli E. Palamidessi A. Scita G. (2013). Membrane and actin dynamics interplay at lamellipodia leading edge. *Curr. Opin. Cell Biol.* 25 565–573. 10.1016/j.ceb.2013.04.001 23639310

[B6] Borovac J. Bosch M. Okamoto K. (2018). Regulation of actin dynamics during structural plasticity of dendritic spines: signaling messengers and actin-binding proteins. *Mol. Cell Neurosci.* 91 122–130. 10.1016/j.mcn.2018.07.001 30004015

[B7] Briatore F. Patrizi A. Viltono L. Sassoè-Pognetto M. Wulff P. (2010). Quantitative organization of GABAergic synapses in the molecular layer of the mouse cerebellar cortex. *PLoS One* 5:e12119. 10.1371/journal.pone.0012119 20711348 PMC2920831

[B8] Bubb M. R. Senderowicz A. M. Sausville E. A. Duncan K. L. Korn E. D. (1994). Jasplakinolide, a cytotoxic natural product, induces actin polymerization and competitively inhibits the binding of phalloidin to F-actin. *J. Biol. Chem.* 269 14869–14871. 8195116

[B9] Cameron R. S. Liu C. Mixon A. S. Pihkala J. P. Rahn R. J. Cameron P. L. (2007). Myosin16b: the COOH-tail region directs localization to the nucleus and overexpression delays S-phase progression. *Cell Motil. Cytoskeleton* 64 19–48. 10.1002/cm.20162 17029291

[B10] Cameron R. S. Liu C. Pihkala J. P. (2013). Myosin 16 levels fluctuate during the cell cycle and are downregulated in response to DNA replication stress. *Cytoskeleton* 70 328–348. 10.1002/cm.21109 23596177

[B11] Chang S. C. Pauls D. L. Lange C. Sasanfar R. Santangelo S. L. (2013). Sex-specific association of a common variant of the XG gene with autism spectrum disorders. *Am. J. Med. Genet. B Neuropsychiatr. Genet.* 162B 742–750. 10.1002/ajmg.b.32165 24132906

[B12] Chazeau A. Garcia M. Czondor K. Perrais D. Tessier B. Giannone G. (2015). Mechanical coupling between transsynaptic N-cadherin adhesions and actin flow stabilizes dendritic spines. *Mol. Biol. Cell* 26 859–873. 10.1091/mbc.E14-06-1086 25568337 PMC4342023

[B13] Chazeau A. Giannone G. (2016). Organization and dynamics of the actin cytoskeleton during dendritic spine morphological remodeling. *Cell Mol. Life Sci.* 73 3053–3073. 10.1007/s00018-016-2214-1 27105623 PMC11108290

[B14] Chazeau A. Mehidi A. Nair D. Gautier J. J. Leduc C. Chamma I. (2014). Nanoscale segregation of actin nucleation and elongation factors determines dendritic spine protrusion. *EMBO J.* 33 2745–2764. 10.15252/embj.201488837 25293574 PMC4282554

[B15] Chen B. Brinkmann K. Chen Z. Pak C. W. Liao Y. Shi S. (2014). The WAVE regulatory complex links diverse receptors to the actin cytoskeleton. *Cell* 156 195–207. 10.1016/j.cell.2013.11.048 24439376 PMC4059610

[B16] Chen J. H. Kellner Y. Zagrebelsky M. Grunwald M. Korte M. Walla P. J. (2015). Two-Photon correlation spectroscopy in single dendritic spines reveals fast actin filament reorganization during activity-dependent growth. *PLoS One* 10:e0128241. 10.1371/journal.pone.0128241 26020927 PMC4447372

[B17] Chen Z. Borek D. Padrick S. B. Gomez T. S. Metlagel Z. Ismail A. M. (2010). Structure and control of the actin regulatory WAVE complex. *Nature* 468 533–538. 10.1038/nature09623 21107423 PMC3085272

[B18] Cingolani L. A. Goda Y. (2008). Actin in action: the interplay between the actin cytoskeleton and synaptic efficacy. *Nat. Rev. Neurosci.* 9 344–356. 10.1038/nrn2373 18425089

[B19] Cramer L. P. (1999). Role of actin-filament disassembly in lamellipodium protrusion in motile cells revealed using the drug jasplakinolide. *Curr. Biol.* 9 1095–1105. 10.1016/s0960-9822(99)80478-3 10531004

[B20] de la Torre-Ubieta L. Won H. Stein J. L. Geschwind D. H. (2016). Advancing the understanding of autism disease mechanisms through genetics. *Nat. Med.* 22 345–361. 10.1038/nm.4071 27050589 PMC5072455

[B21] Dolan B. M. Duron S. G. Campbell D. A. Vollrath B. Shankaranarayana Rao B. S. Ko H. Y. (2013). Rescue of fragile X syndrome phenotypes in Fmr1 KO mice by the small-molecule PAK inhibitor FRAX486. *Proc. Natl. Acad. Sci. U.S.A.* 110 5671–5676. 10.1073/pnas.1219383110 23509247 PMC3619302

[B22] Duffney L. J. Zhong P. Wei J. Matas E. Cheng J. Qin L. (2015). Autism-like deficits in Shank3-Deficient mice are rescued by targeting actin regulators. *Cell Rep.* 11 1400–1413. 10.1016/j.celrep.2015.04.064 26027926 PMC4464902

[B23] Fujita-Jimbo E. Momoi T. (2014). Specific expression of FOXP2 in cerebellum improves ultrasonic vocalization in heterozygous but not in homozygous Foxp2 (R552H) knock-in pups. *Neurosci. Lett.* 566 162–166. 10.1016/j.neulet.2014.02.062 24607928

[B24] Gromova K. V. Muhia M. Rothammer N. Gee C. E. Thies E. Schaefer I. (2018). Neurobeachin and the Kinesin KIF21B are critical for endocytic recycling of NMDA receptors and regulate social behavior. *Cell Rep.* 23 2705–2717. 10.1016/j.celrep.2018.04.112 29847800

[B25] Gross J. A. Pacis A. Chen G. G. Drupals M. Lutz P. E. Barreiro L. B. (2017). Gene-body 5-hydroxymethylation is associated with gene expression changes in the prefrontal cortex of depressed individuals. *Transl. Psychiatry* 7:e1119. 10.1038/tp.2017.93 28485726 PMC5534961

[B26] Grove M. Demyanenko G. Echarri A. Zipfel P. A. Quiroz M. E. Rodriguiz R. M. (2004). ABI2-deficient mice exhibit defective cell migration, aberrant dendritic spine morphogenesis, and deficits in learning and memory. *Mol. Cell Biol.* 24 10905–10922. 10.1128/mcb.24.24.10905-10922.2004 15572692 PMC533973

[B27] Ha S. Lee D. Cho Y. S. Chung C. Yoo Y. E. Kim J. (2016). Cerebellar Shank2 regulates excitatory synapse density, motor coordination, and specific repetitive and anxiety-like behaviors. *J. Neurosci.* 36 12129–12143. 10.1523/jneurosci.1849-16.2016 27903723 PMC6601982

[B28] Hazai D. Szudoczki R. Ding J. Soderling S. H. Weinberg R. J. Sotonyi P. (2013). Ultrastructural abnormalities in CA1 hippocampus caused by deletion of the actin regulator WAVE-1. *PLoS One* 8:e75248. 10.1371/journal.pone.0075248 24086480 PMC3783472

[B29] Hlushchenko I. Khanal P. Abouelezz A. Paavilainen V. O. Hotulainen P. (2018). ASD-Associated de novo mutations in five actin regulators show both shared and distinct defects in dendritic spines and inhibitory synapses in cultured hippocampal neurons. *Front. Cell Neurosci.* 12:217. 10.3389/fncel.2018.00217 30123108 PMC6085419

[B30] Hlushchenko I. Koskinen M. Hotulainen P. (2016). Dendritic spine actin dynamics in neuronal maturation and synaptic plasticity. *Cytoskeleton* 73 435–441. 10.1002/cm.21280 26849484

[B31] Honkura N. Matsuzaki M. Noguchi J. Ellis-Davies G. C. Kasai H. (2008). The subspine organization of actin fibers regulates the structure and plasticity of dendritic spines. *Neuron* 57 719–729. 10.1016/j.neuron.2008.01.013 18341992

[B32] Hotulainen P. Hoogenraad C. C. (2010). Actin in dendritic spines: connecting dynamics to function. *J. Cell Biol.* 189 619–629. 10.1083/jcb.201003008 20457765 PMC2872912

[B33] Hotulainen P. Llano O. Smirnov S. Tanhuanpaa K. Faix J. Rivera C. (2009). Defining mechanisms of actin polymerization and depolymerization during dendritic spine morphogenesis. *J. Cell Biol.* 185 323–339. 10.1083/jcb.200809046 19380880 PMC2700375

[B34] Ito M. (2001). Cerebellar long-term depression: characterization, signal transduction, and functional roles. *Physiol. Rev.* 81 1143–1195. 10.1152/physrev.2001.81.3.1143 11427694

[B35] Joensuu M. Lanoue V. Hotulainen P. (2018). Dendritic spine actin cytoskeleton in autism spectrum disorder. *Prog. Neuropsychopharmacol. Biol. Psychiatry* 84 362–381. 10.1016/j.pnpbp.2017.08.023 28870634

[B36] Johnson H. W. Schell M. J. (2009). Neuronal IP3 3-kinase is an F-actin-bundling protein: role in dendritic targeting and regulation of spine morphology. *Mol. Biol. Cell* 20 5166–5180. 10.1091/mbc.E09-01-0083 19846664 PMC2793293

[B37] Kao C. F. Chen H. W. Chen H. C. Yang J. H. Huang M. C. Chiu Y. H. (2016). Identification of susceptible loci and enriched pathways for Bipolar II Disorder using genome-wide association studies. *Int. J. Neuropsychopharmacol.* 19:yw064. 10.1093/ijnp/pyw064 27450446 PMC5203756

[B38] Kasai H. Fukuda M. Watanabe S. Hayashi-Takagi A. Noguchi J. (2010). Structural dynamics of dendritic spines in memory and cognition. *Trends Neurosci.* 33 121–129. 10.1016/j.tins.2010.01.001 20138375

[B39] Kawabata Galbraith K. Fujishima K. Mizuno H. Lee S. J. Uemura T. Sakimura K. (2018). MTSS1 regulation of actin-nucleating formin DAAM1 in dendritic filopodia determines final dendritic configuration of purkinje cells. *Cell Rep.* 24 95–106.e9. 10.1016/j.celrep.2018.06.013 29972794

[B40] Kengyel A. Becsi B. Konya Z. Sellers J. R. Erdodi F. Nyitrai M. (2015). Ankyrin domain of myosin 16 influences motor function and decreases protein phosphatase catalytic activity. *Eur. Biophys. J.* 44 207–218. 10.1007/s00249-015-1015-z 25775934 PMC8603391

[B41] Kim H. J. DiBernardo A. B. Sloane J. A. Rasband M. N. Solomon D. Kosaras B. (2006). WAVE1 is required for oligodendrocyte morphogenesis and normal CNS myelination. *J. Neurosci.* 26 5849–5859. 10.1523/jneurosci.4921-05.2006 16723544 PMC6675261

[B42] Kim Y. Sung J. Y. Ceglia I. Lee K. W. Ahn J. H. Halford J. M. (2006). Phosphorylation of WAVE1 regulates actin polymerization and dendritic spine morphology. *Nature* 442 814–817. 10.1038/nature04976 16862120

[B43] Kim I. H. Racz B. Wang H. Burianek L. Weinberg R. Yasuda R. (2013). Disruption of Arp2/3 results in asymmetric structural plasticity of dendritic spines and progressive synaptic and behavioral abnormalities. *J. Neurosci.* 33 6081–6092. 10.1523/JNEUROSCI.0035-13.2013 23554489 PMC3656411

[B44] Kneussel M. Wagner W. (2013). Myosin motors at neuronal synapses: drivers of membrane transport and actin dynamics. *Nat. Rev. Neurosci.* 14 233–247. 10.1038/nrn3445 23481482

[B45] Koberstein J. N. Poplawski S. G. Wimmer M. E. Porcari G. Kao C. Gomes B. (2018). Learning-dependent chromatin remodeling highlights noncoding regulatory regions linked to autism. *Sci. Signal.* 11:eaan6500. 10.1126/scisignal.aan6500 29339533 PMC6180319

[B46] Konietzny A. Bar J. Mikhaylova M. (2017). Dendritic actin cytoskeleton: structure, functions, and regulations. *Front. Cell Neurosci.* 11:147. 10.3389/fncel.2017.00147 28572759 PMC5435805

[B47] Korobova F. Svitkina T. (2010). Molecular architecture of synaptic actin cytoskeleton in hippocampal neurons reveals a mechanism of dendritic spine morphogenesis. *Mol. Biol. Cell* 21 165–176. 10.1091/mbc.E09-07-0596 19889835 PMC2801710

[B48] Koskinen M. Bertling E. Hotulainen P. (2012). Methods to measure actin treadmilling rate in dendritic spines. *Methods Enzymol.* 505 47–58. 10.1016/B978-0-12-388448-0.00011-5 22289447

[B49] Koskinen M. Bertling E. Hotulainen R. Tanhuanpaa K. Hotulainen P. (2014). Myosin IIb controls actin dynamics underlying the dendritic spine maturation. *Mol. Cell Neurosci.* 61C 56–64. 10.1016/j.mcn.2014.05.008 24938665

[B50] Lebensohn A. M. Kirschner M. W. (2009). Activation of the WAVE complex by coincident signals controls actin assembly. *Mol. Cell* 36 512–524. 10.1016/j.molcel.2009.10.024 19917258 PMC2818508

[B51] Lee S. E. Kim Y. Han J. K. Park H. Lee U. Na M. (2016). nArgBP2 regulates excitatory synapse formation by controlling dendritic spine morphology. *Proc. Natl. Acad. Sci. U.S.A.* 113 6749–6754. 10.1073/pnas.1600944113 27226294 PMC4914163

[B52] Lin Y. C. Frei J. A. Kilander M. B. Shen W. Blatt G. J. (2016). A subset of autism-associated genes regulate the structural stability of neurons. *Front. Cell Neurosci.* 10:263. 10.3389/fncel.2016.00263 27909399 PMC5112273

[B53] Liu Y. F. Sowell S. M. Luo Y. Chaubey A. Cameron R. S. Kim H. G. (2015). Autism and intellectual disability-associated KIRREL3 interacts with neuronal proteins MAP1B and MYO16 with potential roles in neurodevelopment. *PLoS One* 10:e0123106. 10.1371/journal.pone.0123106 25902260 PMC4406691

[B54] MacGillavry H. D. Song Y. Raghavachari S. Blanpied T. A. (2013). Nanoscale scaffolding domains within the postsynaptic density concentrate synaptic AMPA receptors. *Neuron* 78 615–622. 10.1016/j.neuron.2013.03.009 23719161 PMC3668352

[B55] Matus A. (2000). Actin-based plasticity in dendritic spines. *Science* 290 754–758. 10.1126/science.290.5492.754 11052932

[B56] Matus A. Ackermann M. Pehling G. Byers H. R. Fujiwara K. (1982). High actin concentrations in brain dendritic spines and postsynaptic densities. *Proc. Natl. Acad. Sci. U.S.A.* 79 7590–7594. 10.1073/pnas.79.23.7590 6760199 PMC347386

[B57] Mikhaylova M. Bar J. van Bommel B. Schatzle P. YuanXiang P. Raman R. (2018). Caldendrin directly couples postsynaptic calcium signals to actin remodeling in dendritic spines. *Neuron* 97 1110–1125. 10.1016/j.neuron.2018.01.046 29478916

[B58] Miki H. Suetsugu S. Takenawa T. (1998). WAVE, a novel WASP-family protein involved in actin reorganization induced by Rac. *EMBO J.* 17 6932–6941. 10.1093/emboj/17.23.6932 9843499 PMC1171041

[B59] Miyagi Y. Yamashita T. Fukaya M. Sonoda T. Okuno T. Yamada K. (2002). Delphilin: a novel PDZ and formin homology domain-containing protein that synaptically colocalizes and interacts with glutamate receptor delta 2 subunit. *J. Neurosci.* 22 803–814. 10.1523/jneurosci.22-03-00803.2002 11826110 PMC6758529

[B60] Njoo C. Agarwal N. Lutz B. Kuner R. (2015). The cannabinoid receptor CB1 interacts with the WAVE1 complex and plays a role in actin dynamics and structural plasticity in neurons. *PLoS Biol.* 13:e1002286. 10.1371/journal.pbio.1002286 26496209 PMC4619884

[B61] Nolen B. J. Tomasevic N. Russell A. Pierce D. W. Jia Z. McCormick C. D. (2009). Characterization of two classes of small molecule inhibitors of Arp2/3 complex. *Nature* 460 1031–1034. 10.1038/nature08231 19648907 PMC2780427

[B62] Oberdick J. Smeyne R. J. Mann J. R. Zackson S. Morgan J. I. (1990). A promoter that drives transgene expression in cerebellar purkinje and retinal bipolar neurons. *Science* 248 223–226. 10.1126/science.2109351 2109351

[B63] Okamoto K. Narayanan R. Lee S. H. Murata K. Hayashi Y. (2007). The role of CaMKII as an F-actin-bundling protein crucial for maintenance of dendritic spine structure. *Proc. Natl. Acad. Sci. U.S.A.* 104 6418–6423. 10.1073/pnas.0701656104 17404223 PMC1851051

[B64] Parkinson G. T. Chamberlain S. E. L. Jaafari N. Turvey M. Mellor J. R. Hanley J. G. (2018). Cortactin regulates endo-lysosomal sorting of AMPARs via direct interaction with GluA2 subunit. *Sci. Rep.* 8:4155. 10.1038/s41598-018-22542-z 29515177 PMC5841360

[B65] Patel K. G. Liu C. Cameron P. L. Cameron R. S. (2001). Myr 8, a novel unconventional myosin expressed during brain development associates with the protein phosphatase catalytic subunits 1alpha and 1gamma1. *J. Neurosci.* 21 7954–7968. 10.1523/jneurosci.21-20-07954.2001 11588169 PMC6763852

[B66] Pathania M. Davenport E. C. Muir J. Sheehan D. F. Lopez-Domenech G. Kittler J. T. (2014). The autism and schizophrenia associated gene CYFIP1 is critical for the maintenance of dendritic complexity and the stabilization of mature spines. *Transl. Psychiatry* 4:e374. 10.1038/tp.2014.16 24667445 PMC3966042

[B67] Peter S. Ten Brinke M. M. Stedehouder J. Reinelt C. M. Wu B. Zhou H. (2016). Dysfunctional cerebellar Purkinje cells contribute to autism-like behaviour in Shank2-deficient mice. *Nat. Commun.* 7:12627. 10.1038/ncomms12627 27581745 PMC5025785

[B68] Pils S. Kopp K. Peterson L. Delgado Tascon J. Nyffenegger-Jann N. J. Hauck C. R. (2012). The adaptor molecule Nck localizes the WAVE complex to promote actin polymerization during CEACAM3-mediated phagocytosis of bacteria. *PLoS One* 7:e32808. 10.1371/journal.pone.0032808 22448228 PMC3308951

[B69] Pollard T. D. Borisy G. G. (2003). Cellular motility driven by assembly and disassembly of actin filaments. *Cell* 112 453–465. 10.1016/s0092-8674(03)00120-x 12600310

[B70] Rex C. S. Gavin C. F. Rubio M. D. Kramar E. A. Chen L. Y. Jia Y. (2010). Myosin IIb regulates actin dynamics during synaptic plasticity and memory formation. *Neuron* 67 603–617. 10.1016/j.neuron.2010.07.016 20797537 PMC2929390

[B71] Rizvi S. A. Neidt E. M. Cui J. Feiger Z. Skau C. T. Gardel M. L. (2009). Identification and characterization of a small molecule inhibitor of formin-mediated actin assembly. *Chem. Biol.* 16 1158–1168. 10.1016/j.chembiol.2009.10.006 19942139 PMC2784894

[B72] Rodriguez-Murillo L. Xu B. Roos J. L. Abecasis G. R. Gogos J. A. Karayiorgou M. (2014). Fine mapping on chromosome 13q32-34 and brain expression analysis implicates MYO16 in schizophrenia. *Neuropsychopharmacology* 39 934–943. 10.1038/npp.2013.293 24141571 PMC3924527

[B73] Rotty J. D. Wu C. Bear J. E. (2013). New insights into the regulation and cellular functions of the ARP2/3 complex. *Nat. Rev. Mol. Cell Biol.* 14 7–12. 10.1038/nrm3492 23212475

[B74] Rubio M. D. Johnson R. Miller C. A. Huganir R. L. Rumbaugh G. (2011). Regulation of synapse structure and function by distinct myosin II motors. *J. Neurosci.* 31 1448–1460. 10.1523/JNEUROSCI.3294-10.2011 21273429 PMC3074980

[B75] Rust M. B. Maritzen T. (2015). Relevance of presynaptic actin dynamics for synapse function and mouse behavior. *Exp. Cell Res.* 335 165–171. 10.1016/j.yexcr.2014.12.020 25579398

[B76] Saarikangas J. Kourdougli N. Senju Y. Chazal G. Segerstrale M. Minkeviciene R. (2015). MIM-Induced membrane bending promotes dendritic spine initiation. *Dev. Cell* 33 644–659. 10.1016/j.devcel.2015.04.014 26051541

[B77] Schindelin J. Arganda-Carreras I. Frise E. Kaynig V. Longair M. Pietzsch T. (2012). Fiji: an open-source platform for biological-image analysis. *Nat. Methods* 9 676–682. 10.1038/nmeth.2019 22743772 PMC3855844

[B78] Schneider C. A. Rasband W. S. Eliceiri K. W. (2012). NIH Image to ImageJ: 25 years of image analysis. *Nat. Methods* 9 671–675. 10.1038/nmeth.2089 22930834 PMC5554542

[B79] Schonewille M. Belmeguenai A. Koekkoek S. K. Houtman S. H. Boele H. J. van Beugen B. J. (2010). Purkinje cell-specific knockout of the protein phosphatase PP2B impairs potentiation and cerebellar motor learning. *Neuron* 67 618–628. 10.1016/j.neuron.2010.07.009 20797538 PMC2941980

[B80] Sdrulla A. D. Linden D. J. (2007). Double dissociation between long-term depression and dendritic spine morphology in cerebellar Purkinje cells. *Nat. Neurosci.* 10 546–548. 10.1038/nn1889 17435753

[B81] Sekerkova G. Loomis P. A. Changyaleket B. Zheng L. Eytan R. Chen B. (2003). Novel espin actin-bundling proteins are localized to Purkinje cell dendritic spines and bind the Src homology 3 adapter protein insulin receptor substrate p53. *J. Neurosci.* 23 1310–1319. 10.1523/jneurosci.23-04-01310.2003 12598619 PMC2854510

[B82] Shemiakina I. I. Ermakova G. V. Cranfill P. J. Baird M. A. Evans R. A. Souslova E. A. (2012). A monomeric red fluorescent protein with low cytotoxicity. *Nat. Commun.* 3:1204. 10.1038/ncomms2208 23149748

[B83] Shen B. Zhang W. Zhang J. Zhou J. Wang J. Chen L. (2014). Efficient genome modification by CRISPR-Cas9 nickase with minimal off-target effects. *Nat. Methods* 11 399–402. 10.1038/nmeth.2857 24584192

[B84] Smith-Hicks C. Xiao B. Deng R. Ji Y. Zhao X. Shepherd J. D. (2010). SRF binding to SRE 6.9 in the Arc promoter is essential for LTD in cultured Purkinje cells. *Nat. Neurosci.* 13 1082–1089. 10.1038/nn.2611 20694003 PMC3003596

[B85] Soderling S. H. Guire E. S. Kaech S. White J. Zhang F. Schutz K. (2007). A WAVE-1 and WRP signaling complex regulates spine density, synaptic plasticity, and memory. *J. Neurosci.* 27 355–365. 10.1523/jneurosci.3209-06.2006 17215396 PMC3740594

[B86] Soderling S. H. Langeberg L. K. Soderling J. A. Davee S. M. Simerly R. Raber J. (2003). Loss of WAVE-1 causes sensorimotor retardation and reduced learning and memory in mice. *Proc. Natl. Acad. Sci. U.S.A.* 100 1723–1728. 10.1073/pnas.0438033100 12578964 PMC149900

[B87] Sokolov A. A. Miall R. C. Ivry R. B. (2017). The cerebellum: adaptive prediction for movement and cognition. *Trends Cogn. Sci.* 21 313–332. 10.1016/j.tics.2017.02.005 28385461 PMC5477675

[B88] Sotelo C. (1978). Purkinje cell ontogeny: formation and maintenance of spines. *Prog. Brain Res.* 48 149–170. 10.1016/s0079-6123(08)61021-3 746152

[B89] Spence E. F. Kanak D. J. Carlson B. R. Soderling S. H. (2016). The Arp2/3 complex is essential for distinct stages of spine synapse maturation, including synapse unsilencing. *J. Neurosci.* 36 9696–9709. 10.1523/JNEUROSCI.0876-16.2016 27629719 PMC5039249

[B90] Spence E. F. Soderling S. H. (2015). Actin out: regulation of the synaptic cytoskeleton. *J. Biol. Chem.* 290 28613–28622. 10.1074/jbc.R115.655118 26453304 PMC4661376

[B91] Star E. N. Kwiatkowski D. J. Murthy V. N. (2002). Rapid turnover of actin in dendritic spines and its regulation by activity. *Nat. Neurosci.* 5 239–246. 10.1038/nn811 11850630

[B92] Takenawa T. Suetsugu S. (2007). The WASP-WAVE protein network: connecting the membrane to the cytoskeleton. *Nat. Rev. Mol. Cell Biol.* 8 37–48. 10.1038/nrm2069 17183359

[B93] Thevenaz P. Unser M. (2007). User-friendly semiautomated assembly of accurate image mosaics in microscopy. *Microsc. Res. Tech.* 70 135–146. 10.1002/jemt.20393 17133410

[B94] Tsai P. T. Hull C. Chu Y. Greene-Colozzi E. Sadowski A. R. Leech J. M. (2012). Autistic-like behaviour and cerebellar dysfunction in Purkinje cell Tsc1 mutant mice. *Nature* 488 647–651. 10.1038/nature11310 22763451 PMC3615424

[B95] Wagner W. Brenowitz S. D. Hammer J. A. III (2011a). Myosin-Va transports the endoplasmic reticulum into the dendritic spines of Purkinje neurons. *Nat. Cell Biol.* 13 40–48. 10.1038/ncb2132 21151132 PMC3403743

[B96] Wagner W. McCroskery S. Hammer J. A. III (2011b). An efficient method for the long-term and specific expression of exogenous cDNAs in cultured Purkinje neurons. *J. Neurosci. Methods* 200 95–105. 10.1016/j.jneumeth.2011.06.006 21708190 PMC3407467

[B97] Wang K. Zhang H. Ma D. Bucan M. Glessner J. T. Abrahams B. S. (2009). Common genetic variants on 5p14.1 associate with autism spectrum disorders. *Nature* 459 528–533. 10.1038/nature07999 19404256 PMC2943511

[B98] Wang S. S. Kloth A. D. Badura A. (2014). The cerebellum, sensitive periods, and autism. *Neuron* 83 518–532. 10.1016/j.neuron.2014.07.016 25102558 PMC4135479

[B99] Yamasaki M. Miyazaki T. Azechi H. Abe M. Natsume R. Hagiwara T. (2011). Glutamate receptor δ2 is essential for input pathway-dependent regulation of synaptic AMPAR contents in cerebellar Purkinje cells. *J. Neurosci.* 31 3362–3374. 10.1523/JNEUROSCI.5601-10.2011 21368048 PMC6623914

[B100] Yan Z. Kim E. Datta D. Lewis D. A. Soderling S. H. (2016). Synaptic actin dysregulation, a convergent mechanism of mental disorders? *J. Neurosci.* 36 11411–11417. 10.1523/jneurosci.2360-16.2016 27911743 PMC5125208

[B101] Yang Q. Zhang X. F. Pollard T. D. Forscher P. (2012). Arp2/3 complex-dependent actin networks constrain myosin II function in driving retrograde actin flow. *J. Cell Biol.* 197 939–956. 10.1083/jcb.201111052 22711700 PMC3384413

[B102] Yokoyama K. Tezuka T. Kotani M. Nakazawa T. Hoshina N. Shimoda Y. (2011). NYAP: a phosphoprotein family that links PI3K to WAVE1 signalling in neurons. *EMBO J.* 30 4739–4754. 10.1038/emboj.2011.348 21946561 PMC3243607

[B103] Zhang B. Chen L. Y. Liu X. Maxeiner S. Lee S. J. Gokce O. (2015). Neuroligins sculpt cerebellar purkinje-cell circuits by differential control of distinct classes of synapses. *Neuron* 87 781–796. 10.1016/j.neuron.2015.07.020 26291161 PMC4545494

